# Machine learning-based drug-drug interaction prediction: a critical review of models, limitations, and data challenges

**DOI:** 10.3389/fphar.2025.1632775

**Published:** 2025-07-30

**Authors:** Flaviu-Ioan Gheorghita, Vlad-Ioan Bocanet, Laszlo Barna Iantovics

**Affiliations:** ^1^ Doctoral School of Letters, Humanities and Applied Sciences, George Emil Palade University of Medicine, Pharmacy, Science, and Technology of Târgu Mureş, Târgu Mureş, Romania; ^2^ Department of Manufacturing Engineering, Technical University of Cluj-Napoca, Cluj-Napoca, Romania; ^3^ Department of Electrical Engineering and Information Technology, George Emil Palade University of Medicine, Pharmacy, Science, and Technology of Târgu Mureş, Târgu Mureş, Romania

**Keywords:** drug-drug interaction, adverse drug reactions, machine learning techniques, healthcare, semi-supervised learning, supervised learning, graph-based learning

## Abstract

**Background/Objectives:**

New computational methods, based on statistical, machine learning, and deep learning techniques using drug-related entities (e.g., genes, protein bindings, etc.), help reduce the costs of *in-vitro* experiments through drug-drug interaction prediction (DDIp). This review examines recent advances in DDIp. It presents an in-depth review of the state-of-the-art studies relating to semi-supervised, supervised, self-supervised learning, and other techniques such as graph-based learning and matrix factorization methods for predicting DDIs. All possible interactions between drugs are not known, and accurately predicting interactions is even more difficult due to the complex nature of drug-drug interactions (DDI).

**Methods:**

Of the 49 papers published in Web of Science in the last 6 years, 24 papers were considered relevant based on information presented in their titles and abstracts. The included articles focus specifically on predicting DDIs using a type of machine learning algorithm. Excluded articles focused on drug discovery, drug repurposing, molecular representation, or the extraction of biomedical interactions. The methodology, results limitations, and future research directions were studied for each paper. Common challenges, limitations, and future research directions were analyzed.

**Results and conclusion:**

The main limitations are class imbalance, poor performance on new drugs, limited explainability, and the need for additional data sources.

## 1 Introduction

Drug-drug interactions (DDIs) occur when the pharmacokinetics (how drugs are absorbed, distributed, metabolized, and excreted) or pharmacodynamics (how drugs affect the body) of one drug are altered by the presence of another (Rowland, 2019). This can happen in patients taking multiple medications, whether for a single condition requiring combination therapy, such as cancer or acquired immunodeficiency syndrome (AIDS), or for multiple conditions needing separate treatments. While many drug interactions have no significant clinical impact, those involving drugs with a narrow therapeutic window can lead to serious consequences, including reduced effectiveness or increased toxicity. Intentional interactions are often beneficial, but unintentional interactions can result in ineffective treatment or severe side effects, potentially limiting the use of the drugs involved or even leading to their withdrawal from the market (Rowland, 2019).

First clinical issues recognized due to DDIs were discovered and acknowledged in the early 1960s (Sjöqvist and Böttiger, 2010). At that time, it was a revelation that the drugs can interact with each other, by changing their absorption, metabolism, and renal elimination (ADME), even if the pharmacological notions of synergism, antagonism, and potentiation were already known by pharmacists and chemists.

These reactions occur when two or more drugs are administered together, changing how the drugs affect the body. A DDI can delay, enhance, or decrease the absorption or the therapeutic effect of either/both drugs and can cause an unanticipated side effect (Obach, 2009). The stability and predictability of drugs can also affect DDIs by ensuring consistent drug levels in the bloodstream and minimizing the risk of unexpected interactions (Mircia et al., 2012; 2013).

Rational drug design, also referred to as drug design, involves developing new drugs based on the understanding of biological targets. Using this approach, more effective and safer medications are created by adapting drug properties to interact precisely with specific biological mechanisms (Smith and Williams, 2002).

Kashyap et al. (2013) showed in their study the existence of severe drug-drug interactions, especially among elderly Indian patients, since they have prescriptions for a greater number of drugs. The study was assumed to determine the occurrence of drug interactions as well as their predictors. The DDIs have triggered the withdrawal of some specific high-profile drugs from the market; hence, it is important to evaluate the potential interactions of the drugs before prescribing them.

As more patients take multiple drugs, predicting DDIs becomes more important. Traditional methods, like *in-vitro* and *in-vivo* experiments, are time-consuming, labor-intensive, and often ineffective at measuring DDI-related side effects (Yan et al., 2022). Computational methods, however, offer a cost-effective and highly accurate alternative for predicting new DDIs, making them essential in bioinformatics research and drug development (Yan et al., 2022). The advancement of medical technologies and the growing application of multi-drug treatments further underscore the urgent need for developing these computational approaches to identify potential DDIs efficiently.

Machine learning (ML) techniques have greatly improved the prediction of drug-drug interactions. For example, one study (Hecker et al., 2024) used a deep neural network to predict DDIs and drug-food interactions in patients with multiple sclerosis. In another study (Seo et al., 2023), the researchers trained a deep learning model to predict DDIs by integrating chemical structure similarity and protein–protein interaction information from drug-binding proteins. There are many more examples of how ML algorithms are used in predicting DDIs. In this paper, we analyze semi-supervised, supervised, and some other methods of predicting DDIs with the objective of identifying the methods used in recent studies, what their performance is, and what the limitations and possible future directions of research are. By looking at the methodology of each study, one can determine common patterns or get innovative ideas that might power their research. Understanding the limitations of each proposed method also brings important insights into what is and is not possible using the analyzed methods. Also, coupled with the future directions of each study, researchers can understand where each study ends and how the research can be continued.

The upcoming part of the paper is organized as follows: the second section outlines the methodology used for searching and selecting the papers, as well as the findings of this selection. In the third section, the studies are grouped by method type and analyzed, each paper identifying the methodology that was used, the obtained results, limitations, and future research directions. In the discussion section, we provide an overview of the findings while giving a bird’s-eye view of the research done on learning methods for predicting DDIs. We also look at the limitations of the studies as possible future researches that can be followed by researchers in the field. In the final section, we conclude this review.

### 1.1 Scope and positioning

There have been a number of reviews on the subject of predicting DDIs using ML in the last few years. For example Lin et al. (2023), looked at deep learning and graph-based models and how well they worked on different datasets. Han et al. (2022) gave a more general overview of ML methods, listing the main problems and classifying the methods into broad groups. Zhao et al. (2024) looked at the subject differently by analyzing databases, web tools, and common computational strategies that are used in the field.

While these contributions are significant, our review differs in both scope and structure. Rather than concentrating exclusively on a particular model family or dataset benchmark, we aim to provide a synthesis of multiple learning paradigms—supervised, semi-supervised, self-supervised, and structured methods—and see how these methods work in real life, where data quality and model explainability are often just as important as accuracy. The goal was not only to summarize the methods but also to show where they tend to work best, where they have trouble, and what gaps are still open for future research.

## 2 Methodology

**FIGURE 1:**
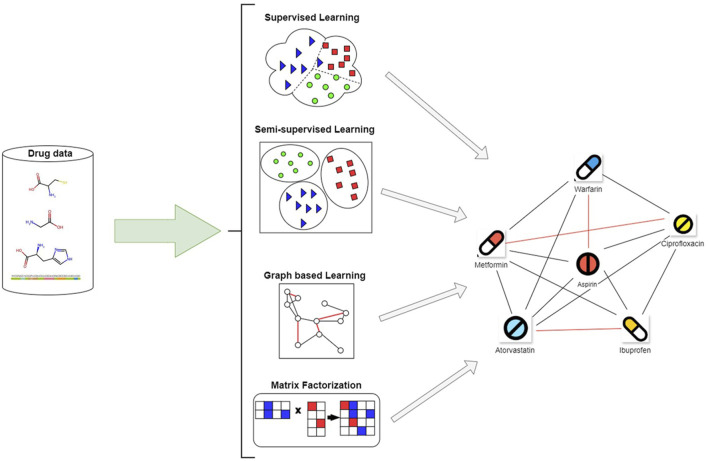
Overview of article selection and classification strategy used in this review.

We chose Web of Science (WoS) due to its curated indexing quality, multidisciplinary scope, and compatibility with advanced citation and bibliometric analysis tools. While databases such as Scopus and Elsevier’s ScienceDirect also provide access to a wide range of ML and pharmacology publications, a significant proportion of high-impact articles indexed in those platforms are also covered by WoS. To maintain methodological consistency and reduce duplication during screening, we prioritized WoS-indexed literature while acknowledging that complementary databases could be incorporated in future systematic extensions of this review. The authors conducted a comprehensive search in the WoS database using the queries: “supervised learning drug-drug interaction”, “semi-supervised learning drug-drug interaction”, “self-supervised learning drug-drug interaction”, “structured learning drug-drug interaction” across all fields (including topic, title, abstract, etc.) with the many variations of: “supervised learning”, “drug-drug interaction”, “DDI”, a.s.o.), as illustrated in Figure 1. The combination of keywords that was used also yielded articles that did not use just supervised methods but also other ML methods or a mix of methods. As a result, even though the studies did not use strictly supervised methods to predict DDIs, the articles were considered relevant and were included in the analysis.

**FIGURE 2:**
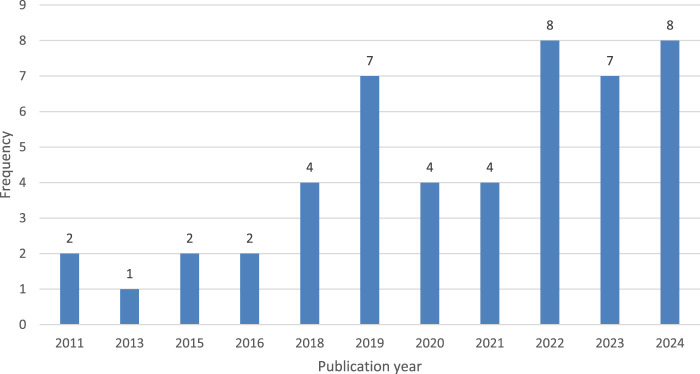
The resulting papers grouped by publication year.

The search yielded a total of 49 unique papers published starting in 2011. Interest in this topic has significantly increased since 2018, with a notable rise in publications over the last 6 years (2018–2024), as illustrated in Figure 2. 42 papers were published in these 6 years, indicating a growing interest in ML methods for DDIp.

**FIGURE 3:**
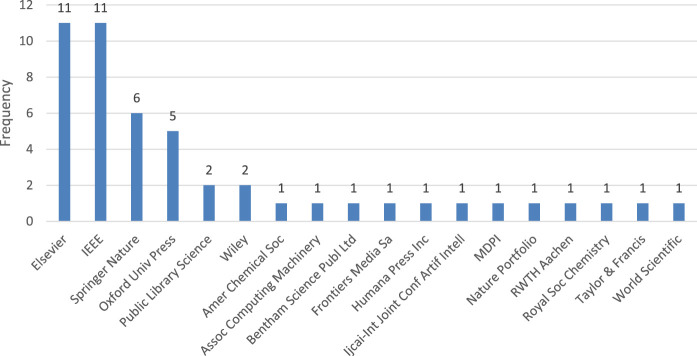
The resulting papers grouped by publisher.

The majority of these papers were published by prominent publishing houses such as Elsevier, IEEE, Springer Nature, and Oxford University Press, as depicted in Figure 3. The rest of the articles were spread out among other publishers.

**FIGURE 4:**
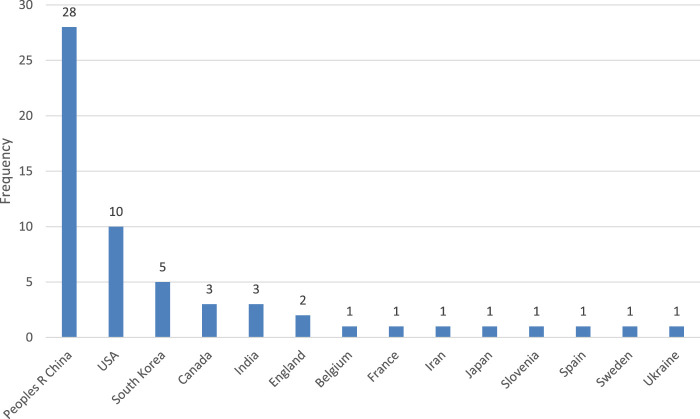
The country of origin of the authors of the publications as per WoS.

Most of the authors are from research institutions located in the People’s Republic of China and the United States of America. According to Web of Science, the country is determined by “countries or regions in author addresses, and not to countries or regions where research studies were conducted” (WOS, 2024), shown in Figure 4.

We assessed titles and abstracts to ensure paper relevance. Only articles that specifically dealt with predicting DDIs with a type of ML algorithm were considered relevant.

Some of the excluded articles focused on drug discovery, drug repurposing, molecular representation, or extraction of biomedical interactions (including DDIs). Papers that did not focus specifically on DDIs were excluded from the analysis. For example, some papers used neural networks to predict molecular structures of drugs, which was useful for drug discovery but not directly for predicting DDIs. Other studies focused on mining DDIs from electronic health records but did not use algorithms for predicting them.

We identified 24 papers highly relevant to ML methods for DDI prediction. One paper did not have a full text available and was excluded from the analysis. The selected papers form the basis of our detailed analysis and review. Supervised, semi-supervised, self-supervised, and other similar learning methods were considered.

## 3 Learning methods for predicting DDIs

Emphasizing the several ML techniques applied in the drug-interaction field, this review article examines recent developments in supervised methods for predicting the DDIs. Each paper is analyzed individually, and an overview of the methodologies employed, their results, and respective strengths and limitations of each is presented below. Three main groups—semi-supervised learning methods, supervised learning methods, and other learning techniques, including self-supervised learning, graph-based learning, and matrix factorization methods—are used to categorize the approaches in the analysis.

### 3.1 Semi-supervised learning methods

Semi-supervised learning techniques enhance model performance using both labeled and unlabeled data. Given the lack of labeled interaction data in DDIp, this method is especially helpful. Semi-supervised learning leverages the abundant unlabeled data to augment the learning process, enhancing the model’s ability to generalize from limited labeled samples.

#### 3.1.1 A meta-learning framework using representation learning to predict drug-drug interaction

The study performed by Deepika and Geetha (2018), integrated representation learning, positive-unlabeled (PU) learning, and meta-learning techniques. The authors used multiple data sources to construct feature networks. These networks serve to represent various drug-related entities such as substructures, target interactions, side-effects, and therapeutic relationships. The Node to Vector (node2vec) algorithm is applied for network embedding to obtain dense, low-dimensional drug representations. The representations are then used by a bagging Support Vector Machine (SVM) classifier to predict drug interactions. This classifier outperforms the traditional one-class SVM by leveraging both labeled (positive) and unlabeled data. The final predictions are refined using a meta-classifier that combines outputs from base classifiers, further enhancing prediction reliability. The framework significantly improves performance, achieving a 22% increase with node2vec and a 12.7% boost with the PU learning approach compared with traditional methods such as logistic regression (LR), decision trees (DT), and k-nearest neighbors. The number of new positive DDIp is relatively low due to the substantial imbalance between positive (3,299 drug pairs) and unlabeled data (149,878 pairs) with a ratio of 1:44. Enhancing the dataset with additional positive DDIs from sources like TwoSides and Kyoto Encyclopedia of Genes and Genomes (KEGG) could improve prediction accuracy. TwoSides (TLab, 2024) is a comprehensive database containing drug-drug-effect relationships with over 3,300 drugs and 63,000 combinations. KEGG, on the other hand, is a database designed to explain high-level functions and utilities of biological systems using molecular-level information, particularly large-scale datasets produced by genome sequencing and other high-throughput experimental technologies (KEGG, 2024). Furthermore, the chemical-based classifier’s performance could be boosted by considering more molecular fingerprints and 3D structures of drugs. Future work could also explore integrating additional drug-related features such as pathways, enzymes, transporters, and gene ontology to enhance the model’s predictive power.

#### 3.1.2 MLRDA: a Multi-Task Semi-Supervised Learning Framework for drug-drug interaction prediction

Chu et al. (2019) integrated multiple drug features and leveraged multi-task learning. Using an unsupervised disentangling loss known as Cumulative Cross-Covariance (CuXCov) in conjunction with a classification loss to separate DDI-relevant from irrelevant ones, the authors created the Multi-Task Semi-Supervised Learning Framework for DDI Prediction (MLRDA) framework. CuXCov improves prediction performance relative to conventional techniques, including logistic regression, decision trees, and k-nearest neighbors, by separating the components of the data representation that help predict DDIs from those that do not. This method is meant to solve the problems with sparse DDI labels and overfitting risk when using several drug features. Drug features are encoded by MLRDA using an autoencoder structure; the aggregation module aggregates predictions from several features using an attention mechanism. The framework was tested on two real-world datasets, C1IT and C2IS, using various drug features such as chemical structures, indications, targets, and side effects. The C1IT dataset includes detailed information on drug indications together with their chemical structures, covering approximately 5,000 drugs, while the C2IS dataset focuses on drug targets and side effects, containing data on around 3,000 drugs, providing a comprehensive basis for interaction predictions. Experimental results demonstrated that MLRDA significantly outperforms state-of-the-art DDIp methods at that time (i.e., Nearest Neighbor, Label Propagation, Dyadic Prediction, Graph AutoEncoder, Deep DDI, Ladder Network), achieving up to a 10.3% improvement in the area under the precision-recall curve (AUPR).

One limitation of Chu et al.’ study is the challenge of effectively incorporating multiple drug features in a multi-task learning model without overfitting, especially given the sparse nature of DDI labels. This complexity increases the risk of overfitting due to the high number of parameters when integrating multiple features. Another limitation is the potential bias brought about by the representation learnt by unsupervised techniques, which might entangle factors relevant to DDI predictions with irrelevant fluctuations, so lowering predictive accuracy. Future directions suggested developing advanced techniques for using unlabeled data to improve prediction accuracy and improving the disentangling mechanisms to better separate relevant from irrelevant aspects. Further research is also required to maximize the batch size for an improved estimate of cross-covariance matrices without sacrificing generalizability.

#### 3.1.3 Designing of information model for prediction of drug-drug interactions based on calculation of target and therapeutic similarity

Initially labeling data using the K-Means clustering algorithm, a semi-supervised approach was used whereby classification with an SVM predicted interactions. Better accuracy was shown by their approach, which produced an area under the curve (AUC) of 98.5 ± 0.05 than by other models with AUCs of 0.968 and 0.912. These findings show that the model forecasts DDIs using biological and therapeutic similarities with efficiency.

Still showing several restrictions is the paper (Marushchak and Kosarevych, 2020). One main restriction is the reliance on DrugBank data, which might not cover all possible DDIs, thus influencing the comprehensiveness of the model. The 54% accuracy of the clustering technique applied for data labeling shows room for development in the data labeling process. Moreover, the work applied a semi-supervised method that might not fully exploit the possibilities of more intricate or alternative ML methods. The authors propose adding more elements to the model, such as enzyme similarity and transporter similarity, to raise the predictive accuracy in next studies. To further generalize the applicability and resilience of the model, they also advise investigating more complex data labeling methods and assessing them against other datasets.

#### 3.1.4 Semi-supervised learning algorithm for identifying high-priority drug-drug interactions through adverse event reports

The study (Liu et al., 2020) used adverse event reports from the FDA’s FAERS database to identify high-priority DDIs. The FDA Adverse Event Reporting System (FAERS) is a database supporting the FDA’s post-marketing safety surveillance for drugs and biologics. It contains adverse event and medication error reports coded with MedDRA terminology and structured according to ICH E2B guidelines (openFDA, n.d.). The authors integrated several data sources, including FAERS, DrugBank, and Drugs.com, which consisted of a combination of labeled and unlabeled data to enhance model performance. DrugBank is a comprehensive bioinformatics and cheminformatics database that contains detailed chemical data with many drugs target information, and contains over 4,100 drug entries and over 14,000 linked protein and drug target sequences. It supports various applications such as *in silico* drug target discovery, drug design, and pharmaceutical education, with search capabilities and hyperlinks to other major databases (Wishart et al., 2006). The approach involves extracting features from adverse event reports, constructing a feature matrix for drug pairs, and assigning class labels based on ONC High-Priority and Non-Interruptive Datasets. A pair of stacked autoencoders is trained separately on positive and negative labeled samples, which then serve as screening tools to identify reliable samples from the unlabeled set via reconstruction errors. These reliable samples are added to the original labeled samples to form an augmented training set for a weighted Support Vector Machine (wSVM) algorithm. The results demonstrate that this method effectively differentiates high-priority from low-priority DDIs, achieving improved classification performance as evidenced by metrics such as the F-measure and AUC score. More specifically, the algorithm selected 719 high-confidence, reliable samples from 35,988 unlabeled samples, significantly boosting the predictive accuracy of the wSVM model.

The primary limitation of this study is the reliance on post-marketing surveillance features, which limits applicability to new drugs or those with few adverse event reports, potentially introducing bias due to confounding factors such as drug indications and patient demographics. Additionally, the study only incorporates data from the FAERS database, excluding other valuable clinical sources like electronic health records (EHRs). The scarcity of labeled training data also restricts the comprehensiveness of the predictive results, highlighting the need for more expert-confirmed labeled samples. Future research could focus on integrating drug property features such as chemical and biological attributes to enhance model accuracy, exploring alternative classifiers like random forest or gradient boosting, and continuously refining the high-priority DDI list and alert mechanisms based on user feedback and retrospective data analysis.

#### 3.1.5 SeHNE: semi-supervised heterogeneous network embedding for drug combination

Semi-supervised Heterogeneous Network Embedding for Drug Combining (SeHNE) is a semi-supervised method used in (Tan and Ma, 2020) study, an approach that builds a comprehensive heterogeneous network by combining drug-drug similarity, drug-target interactions, and protein-protein interaction (PPI). From these networks, the method extracts and learns features using non-negative matrix factorization (NMF), which subsequently finds use in classification and drug combination prediction. The model has the advantage of the joint learning framework. Here, the feature extraction is guided by a classifier, enhancing the discriminative power of the features. In their experimental results, the authors demonstrate that SeHNE outperforms state-of-the-art methods for that time, namely, the Ensemble Prediction framework of Synergistic Drug Combinations–EPSDC (Ding et al., 2019) and gradient tree boosting (GTB) in terms of accuracy, particularly when using a polynomial kernel for the SVM classifier. The paper’s findings suggest that SeHNE’s joint learning approach significantly improves the prediction of synergistic and antagonistic drug combinations (with an AUC around 0.7), highlighting its potential for aiding drug discovery and combination therapy development.

Nevertheless, there are several limitations and suggested future research directions. One notable limitation is the potential time-consuming nature of the current network-based algorithms, which could hinder the application of SeHNE to large-scale networks. Even though SeHNE shows better accuracy than current approaches, there is still space for development by means of algorithm optimization to lower computational complexity and investigation of more effective embedding techniques to manage large data sets. Integration of other kinds of biological data to strengthen the network and maybe enhance prediction performance is another direction that can be advised for next studies.

#### 3.1.6 Predicting drug-drug interactions based on integrated similarity and semi-supervised learning

Yan et al. (2022) proposed a method named Drug-Drug Interaction Information System - Supervised Learning (DDI-IS-SL) that integrates multiple sources of drug information to predict DDIs. The method uses a Regularized Least Squares (RLS) classifier as a semi-supervised model. The feature similarity of drugs was calculated using the cosine similarity on high-dimensional binary vectors representing chemical, biological, and phenotypic data. Additionally, the Gaussian Interaction Profile (GIP) kernel similarity was computed based on known DDIs. These similarities were then combined to form a final similarity measure. For prediction, the RLS classifier computed the interaction possibility scores of the drug pairs. The model’s performance was evaluated using 5-fold and 10-fold cross-validation, as well as *de novo* drug validation, showing superior results compared to other methods with AUC values of 0.9691, 0.9745, and 0.9292, respectively. The study highlights that DDI-IS-SL not only effectively integrates various drug data types but also performs efficiently, demonstrating its practical utility in predicting both known and novel DDIs.

Although DDI-IS-SL is an effective method for predicting potential DDIs, there are areas that need improvement. For instance, more advanced techniques could be used to better integrate chemical, biological, and phenotypic drug data. Additionally, exploring other prediction models, such as deep learning and matrix approximation methods, could enhance the identification of DDIs in future research.

#### 3.1.7 Measuring drug similarity using drug-drug interactions

In the study (Lv et al., 2024) performed in 2024 was introduced a new approach to assessing drug similarity was introduced by focusing on the network structure of DDIs. Their method leveraged both unsupervised and semi-supervised learning techniques. Initially, the study used unsupervised learning methods, specifically spectral and hierarchical clustering, enhanced by t-SNE for dimension reduction, to group drugs based on their interaction profiles. This clustering facilitates the identification of almost monochromatic group-group interactions and the functional annotation of compounds with unknown mechanisms of action (MoA). The authors then implement a semi-supervised learning framework to predict unknown DDIs. This involved constructing an affinity matrix from node similarity measures and applying a network projection method to handle the interaction data. The semi-supervised approach, which integrates known interaction data, surpasses traditional methods that rely on chemical structure or MoA, as demonstrated by improved precision, recall, and F1 scores in the prediction tasks. It can be concluded that the approached network-based similarity measure not only enhances DDI prediction, but also aligns well with MoA similarity, thereby offering a robust tool for drug discovery and combination therapy development.

A summary of the analyzed papers that use semi-supervised methods is presented in Table 1.

**TABLE 1:** Papers that study semi-supervised methods applied for DDIp.

Ref. No.	Article title	Method used	Comparative analysis	Comment
Deepika and Geetha (2018)	A meta-learning framework using representation learning to predict DDI	Meta-learning, Representation Learning, PU learning algorithm	Meta-learning combined with representation learning provides robust DDIp, but the complexity of meta-learning may limit scalability	The meta-learning approach offers a new perspective but requires extensive computational resources
Chu et al. (2019)	MLRDA: A Multi-Task Semi-Supervised Learning Framework for DDIp	MLRDA (Multi-Label Robust Disentangling Autoencoders)	Combines multi-task learning with semi-supervised learning to leverage limited labeled data effectively. Outperforms traditional supervised methods	Emphasizes the value of multi-task frameworks but highlights the need for further validation with larger datasets
Marushchak and Kosarevych (2020)	Designing of Information Model for DDIp based on Calculation of Target and Therapeutic Similarity	SVM and K-means based information model	Focuses on calculating target and therapeutic similarity. Effective but may miss interactions not captured by these similarities	Demonstrates a targeted approach but may benefit from integrating additional types of data
Liu et al. (2020)	Semi-Supervised Learning Algorithm for Identifying High-Priority DDI Through Adverse Event Reports	Stacked autoencoders and weighted SVM	Utilizes adverse event reports for DDIp. Effective for high-priority interactions but may not generalize well to all interactions	Highlights the potential of using adverse event data but underscores the need for comprehensive datasets
Tan and Ma (2020)	SeHNE: Semi-supervised Heterogeneous Network Embedding for Drug Combination	SeHNE (Semi-Supervised Learning, Heterogeneous Network Embedding) model	Uses heterogeneous network embedding to capture complex relationships. Shows improvements over homogeneous models	A strong approach but requires high-quality network data for optimal performance
Yan et al. (2022)	Predicting DDIp based on Integrated Similarity and Semi-Supervised Learning	Integrated Similarity, Semi-Supervised Learning	Integrates multiple similarity measures with semi-supervised learning. Effective but computationally intensive	Integrates various data types effectively but may require optimization for speed
Lv et al. (2024)	Measuring drug similarity using drug-drug interactions	Spectral clustering and hierarchical clustering	Focuses on measuring drug similarity through DDIs. Useful for understanding drug relationships but may miss novel interactions	Provides a foundational approach but needs enhancement for discovering new interactions

### 3.2 Supervised learning methods

In model training, supervised learning techniques draw on a labeled dataset. Although these techniques are simple, high performance depends on large labeled datasets. From classic regression models to sophisticated neural networks with different architectures, supervised learning methods are rather varied.

#### 3.2.1 Predicting potential drug-drug interactions on topological and semantic similarity features using statistical learning

Kastrin et al. (2018) conducted a study that combines topological and semantic similarity features within a statistical learning framework. They first constructed DDI networks from multiple databases, such as DrugBank, KEGG, NDF-RT, SemMedDB, and Twosides. National Drug File Reference Terminology (NDF-RT) by the Veterans Health Administration organizes the VHA National Drug File into a structured format that can be used to model drug characteristics and is utilized in FDA Structured Product Labeling (National Cancer Institute, 2018). The Semantic MEDLINE Database (SemMedDB) is a repository containing semantic predications (subject-predicate-object triples) extracted by SemRep, a semantic interpreter for biomedical texts (SemMedDB, 2024). Topological features used for prediction include common neighbor, Jaccard’s coefficient, Adamic/Adar index, preferential attachment, resource allocation, and their variants, while semantic features contain drug therapeutic-based similarity (ATC), chemical structure-based similarity (CHEM), MeSH-based similarity (MESH), and adverse drug effect-based similarity (ADE). The study used both unsupervised and supervised learning techniques, with the latter using classifiers like decision trees, k-nearest neighbors, support vector machines, random forest, and gradient boosting machines. The results indicate that topological information has a higher predictive power than semantic features. Showcasing their precision, recall, F1 measure, and area under the ROC curve (AUC-ROC), the SVM and gradient boosting machine attained the highest predictive performance. This approach uses a larger data set and a balanced distribution of DDI pairs to overcome constraints in earlier studies, thus offering a stronger framework for DDIp.

The research suggests the need to include semantic relations for more expressiveness and accuracy, since it treats possible interactions as simple co-occurrences rather than significant links. By including a weighting system to reflect the confidence score of every relationship, the research also ignores the weights of links, so treating all interactions equally, which could be improved. To improve predictive accuracy, the suggested future directions call for the integration of genomic covariates and free-text data as well as the development of procedures to separate possible from clinically confirmed interactions. Furthermore, taken into account would be the dynamic character of DDI networks by means of temporal elements. The authors of this work intend to create a web-based application to enable larger access for the research community to their approach and to extend the methodology to other forms of interaction, such as drug-target, drug-disease, or drug-food interactions.

#### 3.2.2 MTMA: multi-task multi-attribute learning for the prediction of adverse drug-drug interaction

In the paper Zhu et al. (2020) the authors present a way of leveraging multi-task learning and multi-attribute (MTMA) data for predicting adverse drug-drug interactions (ADDIs). The MTMA model uses two drug attributes (molecular structure and side effects) to model adverse interactions. Two interpretable tensors are used, one for molecular structure interactions and another for side effect interactions, to determine the mechanisms behind these interactions. The model uses l2,1-norm regularization to enforce sparsity in the predicted attribute matrices. This helps to identify molecular substructures and significant side effects for specific ADDIs. The MTMA optimization is done by alternatively using an algorithm based on low-rank tensor decomposition and stochastic gradient descent. From the experimental results, MTMA significantly outperforms nine baseline methods and their variants.

The baseline models used in the evaluation are: a large-scale model that measures Molecular Structure Similarities between two drugs (MSSA) (Vilar et al., 2012), Label propagation Prediction of drug-drug interactions based on clinical Side Effects (LPSE) (Zhang et al., 2015), Computational Prediction based on drugs Functional Similarities (CPFS) (Ferdousi et al., 2017), Multi-task Dyadic regression Model (MDM) (Jin et al., 2017), Deep Learning Method (DLP) (Ryu et al., 2018), Topological and Semantic Similarity Learning method (TSSL) (Kastrin et al., 2018), heterogeneous network-assisted inference framework (MLMA) (Cheng and Zhao, 2014), Similarity-based Model for predicting Large Scale ADDI(SMLS) (Vilar et al., 2014), Sparse Feature Learning ensemble method with Linear Neighborhood regularization (SFLLN) (Zhang et al., 2019). The variants used in the evaluation either did not consider the supervision of attributes (MTMA-S), did not consider self-representation of attributes (MTMA-R), or did not explore leading molecular substructures and side effects (MTMA-E) (Zhu et al., 2020). The MTMA model achieved an AUC of 0.9247 and an AUPR of 0.7515, showing superior predictive performance and ability to discover adverse mechanisms.

MTMA can sometimes be outperformed by the SFLLN algorithm in terms of AUPR, suggesting a need for further refinement. Future research could focus on exploring the intrinsic properties of predicted attribute matrices, such as orthogonality, low-rank, and positive semi-definite properties. The contribution of different attributes to the modeling of ADDI can be done using more sophisticated integration strategies. The paper suggests that incorporating more drug attributes beyond molecular structure and side effects, such as targets, enzymes, and pathways, could improve the comprehensiveness and accuracy of predictions.

#### 3.2.3 Integrated random negative sampling and uncertainty sampling in active learning improve clinical drug safety, drug-drug interaction information retrieval

Xie et al. (2021) used an active learning (AL) approach to enhance the performance of DDI information retrieval from PubMed abstracts. The four AL techniques the authors looked at were traditional AL, traditional AL with random negative sampling, AL with two separate ML algorithms integrated with random negative sampling, and AL with two separate ML algorithms integrated with both random negative sampling and validation sample updates. Both SVM and LR algorithms were used. The integration of random negative sampling is cost-effective as it does not require manual curation and the updating of both training and validation datasets to mitigate sampling biases. The outcome displayed substantial improvements in the precision, especially in the second round of training. For example, for the manually labeled negative samples, the precision increased from 0.45 to 0.83 using SVM. Similarly, for the random negative samples, from 0.70 to 0.82. LR also showed similar trends. This technique improved the retrieval of clinically relevant DDI abstracts by efficiently addressing the problems of imbalanced data and biassed sampling.

Despite the fact it combines uncertainty sampling and random negative sampling, the proposed AL method still has potential for performance and generalizability improvement. Future research could incorporate several natural language processing (NLP) approaches and investigate their application across several DDI knowledge domains to raise effectiveness and robustness.

#### 3.2.4 MDDI-SCL: predicting multi-type drug-drug interactions via supervised contrastive learning

Multi-type Drug-Drug Interaction - Supervised Contrastive Learning (MDDI-SCL) developed by Lin et al. (2022), uses supervised contrastive learning based on a three-module framework: a drug feature encoder using a mean squared error (MSE) loss, a drug latent feature fusion module with supervised contrastive loss, and a multi-type DDI prediction module with classification loss. While the feature fusion module aggregates latent features of drug pairs, optimizing them by supervised contrastive learning to improve classification performance, the drug feature encoder learns low-dimensional drug representations using a multi-head self-attention mechanism and an autoencoder. Evaluated on two datasets and three tasks, the model showed either better or equivalent performance to state-of- the-art approaches like Generative Adversarial Network for Drug-Drug Interaction (GAN-DDI) and Deep Drug-Drug Interaction (DeepDDI). On the first dataset, MDDI-SCL, for instance, exceeded other models in terms of AUPR and accuracy across several tasks by achieving an AUPR of 0.9782 and an accuracy of 0.9378 for predicting of unseen interaction types between known drugs. Furthermore, ablation tests verified the efficiency of supervised contrastive learning and case studies verified the practical relevance of the model by pointing up fresh possible DDIs.

One significant limitation is the unbalanced DDI datasets, leading to poor performance in predicting rare interaction types. Additionally, the current methods, including MDDI-SCL, tend to perform well in predicting interactions between known drugs but often struggle with new drugs. Furthermore, the hyperparameters used in the model were not optimized across all datasets and tasks, which may have affected performance. Future research could focus on refining these hyperparameters and exploring advanced strategies like knowledge graph integration to improve the robustness and applicability of these models.

#### 3.2.5 DeConDFFuse: predicting drug-drug interaction using joint deep convolutional transform learning and decision forest fusion framework

Gupta et al. (2023) used Deep Convolutional Network for Drug Feature Fusion (DeConDFFuse), a supervised deep learning approach, combined with decision forests. This utilizes a representation learning architecture, which engages a convolutional transform learning (CTL) in order to provide categorical and interpretable features for every medication pair. These features are then processed through a multi-channel architecture, where bioactivity descriptors generated by the Signaturizer tool are used as input. The generated features are integrated and refined by a decision forest predictor, ensuring an efficient and robust end-to-end learning process. The dataset used for training and evaluation comes from Stanford’s Biosnap, comprising 1,514 drugs and 48,514 interactions. In comparative evaluations against state-of-the-art methods like Knowledge Graph Neural Network (KGNN) (Lin et al., 2020), Convolutional Long Short-Term Memory (Conv-LSTM), and Graph Embedding DDI, the proposed method demonstrates better performance in key metrics such as precision, AUC-ROC, and AUPRC. Although Graph DDI achieved higher accuracy, F1-score, and recall, the authors’ method does a great job in predicting known-to-interact interactions, which can be used for identifying potential adverse reactions. Emphasizing its adaptability against feature unavailability issues, the study describes the capacity of the method to manage a wide spectrum of bioactivity descriptors and hence relates to drugs with inadequate historical information.

The higher proportion of false positives in this study compared to Graph DDI highlights the need for improving specificity in prediction results as one of its primary limitations. The main focus of the forthcoming studies should be on lowering these false positives, thus enhancing the dependability of the method. The present method is meant to control interactions between pairs of drugs; yet, in reality, many times, combinations of several drugs are involved. Therefore, extending the model to include several medication interactions concurrently shows a major direction for future research. Broadening the use of their framework, another study topic consists in the architecture adaptation for additional biological interaction problems, including drug-target prediction, protein-protein interaction, and drug repositioning.

#### 3.2.6 Evaluating the performance of machine-learning regression models for pharmacokinetic drug-drug interactions

Gill et al. (2023) uses regression-based ML models in order to predict the changes in the drug exposure due to DDIs. The data for the training was collected from 120 clinical DDI studies. This included drug characteristics such as: structure, physicochemical properties, and cytochrome P450 (CYP450) metabolic activity. The study involved RF, elastic net, and support vector regression (SVR) models. The performance has been measured via 5-fold cross-validation. The SVR model demonstrated the highest performance, with 78% of predictions within 2-fold of observed exposure changes. It was found that models using early drug discovery features, particularly CYP450 activity and fraction metabolized data, could predict changes in drug exposure with reasonable accuracy. Significant limitations involve the underestimation of more potent inhibition cases, likely related to the lack of features detailing mechanisms like transporter-mediated DDI, as well as the skewed distribution of sample classes that biased the model towards predicting values within more prevalent data ranges.

Additionally, there are limitations on the small sample size related to the feature set, which raises concerns about overfitting, despite nested cross-validation being employed to mitigate this. Research on the transporter effects’ integration and the use of rectifying techniques for class imbalance techniques, such as synthetic minority oversampling, could be conducted in future studies. Another study could involve validating the model on datasets from different sources to confirm generalizability and applying deep learning algorithms to potentially improve prediction accuracy.

#### 3.2.7 Co-attention graph pooling for efficient pairwise graph interaction learning

The study (Lee et al., 2023) introduces the CAGPool method, which uses co-attention mechanisms to predict DDIs and evaluate graph similarity. The method uses graph convolution networks (GCNs) to represent each drug as a graph, embedding the nodes as atoms and the edges as chemical bonds. Then, a co-attention mechanism is applied to dynamically generate node scores based on the interaction between pairs of graphs. This interaction-aware pooling method reduces computational complexity. The most relevant nodes for the interaction representation are selected, which draws the focus on the significant subgraphs. The experimental evaluation demonstrates that CAGPool outperforms several baseline models–concatenated features method, the Decagon method, Message Passing Neural Network (MPNN) with Concatenation-based Feature Aggregation (MPNN-Concat), Late-Outer, Context-Aware DDI (CADDI), Multi-Head Context-Aware DDI (MHCADDI) –, including those leveraging additional features such as protein-protein interactions, by only using the structural information of drug compounds. The results show superior performance across various metrics, including AUROC, AUPRC, and AP@50 on the Decagon dataset for DDI prediction and MSE, Spearman’s rank correlation coefficient, and Kendall’s rank correlation coefficient for graph similarity tasks.

A limitation identified by this study is that it does not analyze the extracted subgraphs and understand the functional groups (subgraphs) related to specific side effects in DDI. These are challenging to interpret due to their complex biological pathways. For future research, the authors suggest that their method can facilitate further studies by identifying subgraphs that are likely related to functional groups responsible for side effects between drugs. The paper also hints at extending the current model to a graph transformation version. This could include all nodes in the final aggregated clusters without information loss. This would address the issue of discarding nodes during the pooling process. These directions imply a deeper exploration of subgraph functionalities and even improvements in graph pooling techniques.

All the analyzed papers in this section using supervised methods are presented in Table 2 in chronological order.

**TABLE 2:** Papers that study supervised methods of learning for DDIp.

Ref.	Article title	Method used	Comparative analysis	Comment
Kastrin et al. (2018)	Predicting potential DDI on topological and semantic similarity features using statistical learning	Supervised link prediction model with topological and semantic similarity features	Outperforms unsupervised approaches and has better prediction performance on large-scale DDI networks compared to models based solely on topological features	Highlights topological information importance over semantic information
Zhu et al. (2020)	MTMA: Multi-task multi-attribute learning for the prediction of adverse DDI	Multi-Task Multi-Attribute (MTMA) model	Integrates multiple drug attributes and uses tensors for uncovering underlying mechanisms of adverse DDIs, unlike prior black-box models	Also uses l2,1-norm regularization
Xie et al. (2021)	Integrated Random Negative Sampling and Uncertainty Sampling in Active Learning Improve Clinical Drug Safety DDI Information Retrieval	Active Learning method with random negative sampling and uncertainty sampling (SVM and LR)	Better precision and recall over traditional active learning approaches; updates training and validation data and combines random negative sampling with uncertainty sampling	Improves the retrieval of clinically relevant DDI toxicity abstracts from PubMed, and addresses biased sample sets and unbalanced data in large-scale text mining tasks
Lin et al. (2022)	MDDI-SCL: predicting multi-type DDIs via supervised contrastive learning	Multi-type Drug-Drug Interaction Supervised Contrastive Learning	Better prediction accuracy and robustness in multi-type DDI prediction than DeepDDI and MDF-SA-DDI	Uses focal loss and label smoothing to address data imbalance and small sample sizes
Gupta et al. (2023)	DeConDFFuse: Predicting drug-drug interaction using joint deep convolutional transform learning and decision forest fusion framework	DeConDFFuse - a combination of deep convolutional transform learning (DeConFuse) and a decision forest (DF)	Better AUC-ROC and precision than KGNN and Graph Embedding DDI, through joint optimization of feature extraction and classification	Efficiently identifies known-to-interact drug pairs and addresses feature redundancy and non-unique representations found in CNN-based models
Gill et al. (2023)	Evaluating the performance of machine-learning regression models for pharmacokinetic drug-drug interactions	Support Vector Regression (SVR), Random Forest, Elastic Net	SVR model outperforms other regression models, including Random Forest and Elastic Net	Early pharmacokinetic data (i.e., CYP activity and fraction metabolized) are effective predictors in regression models
Lee et al. (2023)	Co-Attention Graph Pooling for Efficient Pairwise Graph Interaction Learning	Co-Attention Graph Pooling (CAGPool) for pairwise graph interaction learning	Outperforms state-of-the-art models (e.g., MPNN-Concat, SimGNN) by capturing graph-level interactions	It reduces computational complexity by focusing on graph-level rather than node-level interactions

#### 3.2.8 Data sources and preprocessing strategies

The preparation techniques, provenance, and extent of the used datasets differ greatly among the investigated studies. Most research depends on publicly available sources, including DrugBank, TWOSides, KEGG, NDF-RT, and Decagon, each of which presents different angles on DDIs, as seen in Table 3. For example, although DrugBank offers complete pharmacokinetic and pharmacodynamic data, TWOSides concentrates especially on negative medication interactions. Graph-based approaches often use Biosnap and Decagon datasets because of their ordered presentation of DDIs. The modeling paradigm also influences the preprocessing methods.

**TABLE 3:** Summary of data sources used in the supervised models analyzed.

Study	Primary data source	Number of drugs	Number of interactions	Key preprocessing steps
Topological and Semantic Features	DrugBank, KEGG, NDF-RT, SemMedDB, TWOSIDES	5.7M (DrugBank)	19K (Twosides)	Graph construction, feature selection
MTMA	TWOSIDES, DrugBank, SIDER	555	576K	Multi-task feature encoding
Active Learning and Uncertainty Sampling	PubMed abstracts, Clinical DDI corpus	600 (positive) + 400 (negative)	N/A	NLP preprocessing, negative sampling
MDDI-SCL	DrugBank	1,258	323K	Supervised contrastive learning
DeConDFFuse	Biosnap DDI dataset	1,059	48K	Convolutional feature transformation
PK-DDI Regression	Washington Drug Interaction Database, SimCYP	120 clinical studies	N/A	Regression model training
CAGPool	Decagon	4.5M (total)	964 DDI types	Graph neural network transformation

### 3.3 Self-supervised methods

Self-supervised learning methods generate labels from the data itself, often using pretext tasks to learn useful representations.

#### 3.3.1 Multidrug representation learning based on pretraining model and molecular graph for drug interaction and combination prediction

The paper Ren et al. (2022) presents a method for DDIp and drug combinations that uses a multidrug representation learning framework named Molecular Graph Pretraining for Drug Representation (MGP-DR). The MGP-DR model integrates a large amount of unlabeled drug molecular graph and target information, employing self-supervised learning strategies to mine contextual information within and between drug molecules. This method uses two pretraining tasks: mask atoms prediction, which involves masking and predicting atoms in the molecular graph, and SAB (Separation A-B) score prediction, which measures the network proximity of drug-target modules A and B within a human protein-protein interaction network. The two tasks help learn context-sensitive atomic representations and global drug pair representations. The pretrained transformer encoder is then fine-tuned with a two-layer fully connected neural network for DDI and drug combination prediction.

According to Ren et al. (2022), MGP-DR was compared with multiple state-of-the-art methods including: logistic regression (LR), natural product, mol2Vec, Molecular Variational Autoencoder (MolVAE), DeepDDI, ChemicAl SubstrucTurE Representation (CASTER), Graph Convolutional Network with Bond-aware Message Propagation (GCN-BMP), Link Prediction model based on Multifeature Fusion (LPMF), Link Prediction with Subgraph, Structural Equivalence, and Optimal Structural Equivalence (LP-Sub/SE/OSE), Siamese Sequence-Projection Multi-Layer Perceptron (SSP-MLP), Genetic Algorithm-based method, Neural Fingerprint (NFP), Graph Isomorphism Network (GIN), Complex Embeddings for Simple Link Prediction (ComplEx), Knowledge Base Adversarial Network (KBGAN), Simple Embedding (SimplE), Relational Rotational Embedding (RotatE), a. s.o. The MGP-DR managed to outperform the other methods across multiple metrics (ROC-AUC, PR-AUC, F1, ACC). The model also shows strong potential for DDI predictions, validated through multiple experimental setups.

The paper mentions several limitations and future research directions. Firstly, the current MGP-DR model only performs a simple linear combination of losses, which corresponds to different drug pretraining tasks. This indicates that more sophisticated methods could potentially improve performance. The paper also suggests that incorporating multitask learning strategies during the training phase could improve the model’s ability to learn different aspects of drug interactions from a large number of unlabeled drugs. One other potential area for future research is extending the model to higher-order drug representation learning, which could support combination therapies better.

#### 3.3.2 Predicting drug-drug adverse reactions via multi-view graph contrastive representation model

The authors of this study (Zhuang et al., 2023a) propose the DMVDGI, a self-supervised multi-view graph learning framework designed to predict drug-drug adverse reactions (DDADRs). In this method, multiple biomedical views—such as enzyme, indication, side effect, and transporter data—are used to create comprehensive drug feature representations. A signed network is used to capture positive and negative drug interactions. Contrastive learning is then used to optimize the model by maximizing mutual information between local and global representations. The model was then evaluated on three benchmark datasets: Decagon (a multimodal graph containing protein-protein interactions, drug-protein target interactions, and polypharmacy side effects, represented as DDI with each side effect as a unique edge type) (Zitnik et al., 2018), CRDs (dataset with common CYP-related DDIs containing 807 drugs and 10,106 interactions, with embedded biomedical views including ATC code, molecular fingerprint, protein-based, and target-based information for drug representation) (Rohani and Eslahchi, 2019), and NCRDs (dataset with non-CYP-related DDIs with 807 drugs and 45,737 interactions, and also includes ligand-based, pathway-based, side effect-based, and target-based information for drug representation) (Rohani and Eslahchi, 2019). The baseline models used in the comparison are: GAT (applies the attention mechanism to homogeneous graphs for link prediction tasks) (Veličković et al., 2018), HAN (uses node-level attention and semantic-level attention to capture information from all meta-paths for link prediction tasks) (Wang et al., 2019), MBS - model based on similarity (a fully connected neural network model for prediction tasks using similarity calculation for feature representation) (Kumar and Sharma, 2022), RANEDDI (a relation-aware network embedding model for link predictions integrating relation-aware network structure information) (Yu et al., 2022a), DANN-DDI (a deep attention neural network model for DDADRs prediction that concatenates learned drug embeddings and uses an attention neural network for drug-drug pair representations) (Liu et al., 2023), BiGI (performs link prediction by relating local and global representations) (Cao et al., 2021), SLiCE (learns contextual node representations using localized attention and global information from the entire graph before conducting link prediction experiments) (Wang et al., 2021), DMGI (maximizes agreements between specific node embedding and graph-level summary representation in each relation graph for link prediction) (Park et al., 2020), SUGAR (uses a reinforcement learning algorithm to adaptively select and learn discriminative representations of subgraphs for high-quality feature representation before performing link prediction) (Sun et al., 2021), and Subg-Con (contrasts node embeddings and their context subgraphs to learn regional graph structure information for feature representation, and then performs link prediction) (Jiao et al., 2020). Results show that DMVDGI is significantly better than the baseline models and has achieved the highest AUROC of 0.93182. It also showed robust performance across various metrics, including AUPRC and F1 scores. The study highlights the model’s ability to learn high-quality drug representations with minimal reliance on labeled data.

The excessive inclusion of view information can introduce noise and reduce model performance, and is perceived as a limitation of this method. Also, while the model shows strong results, there is an over-reliance on the similarity threshold in constructing the drug-drug interaction signed network (DDISN). This can potentially lead to sparsity or noise issues. The model also has trouble optimizing hyperparameters such as the number of biomedical views and the GCN layers. Another limitation is the over-reliance on labeled data, which is scarce and valuable. For future research, the authors suggest improving the negative sampling strategy. This can be a promising direction for self-supervised graph representation learning. Additionally, they propose integrating more features from extensive views to enhance the robustness of the model further.

#### 3.3.3 Adaptive dual graph contrastive learning based on a heterogeneous signed network for predicting adverse drug reaction

The methods utilized in the study involve the development of an Adaptive dual graph contrastive learning method (ADGCL). This approach uses heterogeneous signed networks to predict adverse drug reactions (ADRs). It explicitly models positive and negative DDIs using a heterogeneous signed network, which helps in learning rich semantic drug feature representations. A dual graph contrastive learning strategy is used that contains a hybrid of self-supervised contrastive learning and micro-supervised learning to capture high-level features. An adaptive negative sampling method generates high-quality negative samples, and an encoder based on implicit graph neural networks (IGNNs) is designed to capture long-range dependencies in the network. This improves the drug feature representation. The ADGCL method, as a result, combines self-supervised learning with a small degree of supervision. The model was compared with several baseline models: BPNN (Back Propagation Neural Network) (Rumelhart et al., 1986), XGboost (eXtreme Gradient Boosting) (Chen and Guestrin, 2016), GAT (Graph Attention Network) (Veličković et al., 2018), HAN (Heterogeneous Graph Attention Network) (Wang et al., 2019), KGNN (Lin et al., 2020), DeepDDI (Ryu et al., 2018), STNN-DDI (a Substructure-aware Tensor Neural Network) (Yu et al., 2022b), DDIMDL (a multi-modal deep learning framework) (Deng et al., 2020), DM-DDI (deep fusion integrated drug features and topological connections) (Kang et al., 2022), deepMDDI (a deep graph convolutional network framework) (Feng et al., 2022). The results from comprehensive experiments on real-world datasets (Decagon (SNAP, 2024), DrugBank, SIDER (Side Effect Resource) (Kuhn et al., 2016)) demonstrate that ADGCL outperforms the baseline methods, showing significant improvements in AUROC, AUPRC, and F1 scores (Zhuang et al., 2023b).

The authors acknowledge that they had challenges with insufficient data and excessive time complexity. Future studies plan to incorporate more clinical information into the model and use optimization algorithms to enhance its efficiency. Further research can be done on improving the model’s interpretability. This aims to make the model more robust and practical for real-world applications.

#### 3.3.4 Learning self-supervised molecular representations for drug-drug interaction prediction

Kpanou et al. (2024) used in their study a two-step framework leveraging self-supervised contrastive learning to predict DDIs. Their proposed method, self-supervised molecular representation for DDI (SMR-DDI), first involved pre-training a 1D convolutional neural network (CNN) on a large dataset of SMILES strings derived from the ChEMBL22 database. In drug development, ChEMBL is a bioactivity database including special chemical entities, bioactivity measurements, pharmacokinetic data, and pharmacological targets (Ferdousi et al., 2017). In pre-training, they maximized the similarity between many enhanced images of the same molecule using a contrastive loss function, thereby decreasing the similarity between distinct molecules. This way, they created a robust molecular feature space. This pre-trained model served as a feature extractor in the second step, where a feed-forward neural network was trained on the DrugBank dataset to classify the side effects of drug pairs. The approach uses various strategies to handle class imbalance in the data, including batch balancing and weighted random sampling. SMR-DDI achieved competitive performance across multiple tasks, particularly in scenarios involving known drug interactions, but showed slightly reduced efficacy in predicting interactions involving new drugs. The results highlighted the model’s ability to effectively use limited data through data augmentation.

Nevertheless, the reliance on a relatively low-dimensional feature space (262 dimensions), which may not capture the full complexity of molecular interactions compared to higher-dimensional models like Extended-Connectivity Fingerprint (ECFP) or ChemBERT trained on 77 million SMILES strings (ChemBERTa-77M), can be regarded as a limitation of the study. This lower dimensionality could explain the model’s slightly lower performance on certain tasks, particularly those involving interactions with new drugs. Another limitation is the imbalance in the dataset, which requires techniques like batch balancing and weighted random sampling to improve model performance. Future research could explore increasing the dimensionality of the feature space and using a larger, more diverse training dataset. A propagation algorithm for SMILES enumeration could be developed. It can potentially improve the quality of vector representations. Finally, the model’s performance on predicting interactions involving new drugs remains an area for improvement.

#### 3.3.5 Detecting side effects of adverse drug reactions through drug-drug interactions using graph neural networks and self-supervised learning

The paper Chandra Umakantham et al. (2024) presents a framework that uses GNNs and self-supervised learning to predict side effects of ADR due to DDIs. In their approach, drugs are modeled as molecular graphs where atoms are nodes and bonds are edges, capturing the spatial and physical properties of the drugs. A dual-input graph neural network with a 2-stage training phase was developed. Each reactant was pre-trained using a Variational Graph Autoencoder (VGAE) to create a knowledge base. This framework was then trained and tested on the TwoSIDES Polypharmacy Dataset, achieving results with a precision of 75% and an accuracy of 90%. The model was further validated on the DrugBank dataset, showing excellent results with precision, F1, and accuracy of 99%.

One primary limitation of this study is the complexity versus interpretability of the model. The complex model is difficult to interpret due to the architecture of the framework that uses multiple stages and GNNs to model chemical interactions. Also, the study relies on a single dataset, the TwoSIDES Polypharmacy Side Effects Dataset. This is a limitation because it may introduce potential biases and the inability to generalize across different distributions. The authors suggest training and testing on additional datasets, which might enhance the model’s reliability and generalizability. Furthermore, although the dual-path GNN demonstrated stable training, the inherent instability in training multi-path graph neural networks remains a challenge, leading to a need for more robust training techniques. Future research directions include developing more interpretable models, expanding the framework to include additional datasets, and improving the stability and generalizability of the multi-path training framework.

Table 4 provides a comparative overview of key studies that apply to self-supervised learning for DDI prediction. It summarizes the data sources, methods used, and model architectures, highlighting methodological diversity.

**TABLE 4:** Analysis of the papers presenting self-supervised methods of DDIp.

Ref.	Article title	Method used	Comparative analysis	Comment
Ren et al. (2022)	Multidrug representation learning based on pretraining model and molecular graph for drug interaction and combination prediction	MGP-DR (Molecular Graph Pretraining for Drug Representation)	Outperforms state-of-the-art models (e.g., CASTER, GCN-BMP, DEEPDDI) with better accuracy across multiple drug-related prediction tasks	Leverages self-supervised learning and molecular graph-based pretraining
Zhuang et al. (2023a)	Predicting ADEreactions via multi-view graph contrastive representation model	DMVDGI (Drug Multi-View Deep Graph Infomax)	Better AUROC score than state-of-the-art models (e.g., Subg-Con, DANN-DDI) and several supervised models	Integrates multiple biomedical views and applies a signed network for drug interaction representation
Zhuang et al. (2023b)	Adaptive dual graph contrastive learning based on heterogeneous signed network for predicting adverse drug reaction	ADGCL (Adaptive Dual Graph Contrastive Learning)	Superior AUROC and AUPRC comapred with baseline models (DeepDDI, KGNN)	Leverages a heterogeneous signed network and dual contrastive learning while reducing dependency on labeled data with robust performance
Kpanou et al. (2024)	Learning self-supervised molecular representations for DDIp	SMR-DDI (Self-supervised Molecular Representation for DDIp)	Comparable or superior results to state-of-the-art molecular representation methods (e.g., ChemGPT, Mol2Vec, GIN-based models)	Training on smaller datasets and potential for generalizing to new drugs and drug interactions by pre-training on diverse, large-scale unlabeled datasets
Chandra Umakantham et al. (2024)	Detecting Side Effects of ADRs Through DDI Using GNNs and Self-Supervised Learning	GNNs with self-supervised learning	State-of-the-art results on the TwoSIDES Polypharmacy Dataset, while surpassing other methods without molecular structures or GNNs for side-effect prediction	The model was also validated on the DrugBank dataset.

### 3.4 Structured learning methods

#### 3.4.1 Graph-based learning methods

Another large category of learning methods that were studied in the analyzed papers was graph-based learning methods. They model the interactions between drugs as a graph, using GNNs or other graph-based techniques to predict DDIs.

##### 3.4.1.1 Predicting combinations of drugs by exploiting graph embedding of heterogeneous networks

Building on the advancements introduced in the semi-supervised heterogeneous network embedding (SeHNE) framework by Tan and Ma (2020), the study by Song et al. entitled “Predicting combinations of drugs by exploiting graph embedding of heterogeneous networks,” (2022) (Song et al., 2022) further enhances the methodology by emphasizing the topological structure of heterogeneous networks. The previous study by Tan and Ma (2020), based on the SeHNE framework, leveraged non-negative matrix factorization (NMF) within a semi-supervised learning context, integrating drug-drug similarity, drug-target interactions, and protein-protein interaction (PPI) networks. In contrast, the 2022 study by Song et al. (2022) refines this approach by incorporating regularization techniques to preserve local topological structures and integrating Anatomical Therapeutic Chemical (ATC) similarity to capture therapeutic and functional similarities between drugs. This joint learning framework, guided by support vector machines, significantly enhances the discriminative power of the features, resulting in improved prediction accuracy across various metrics, including AUC and average precision.

The (Song et al., 2022) study focuses on graph embedding techniques and introduces new similarity measures. It demonstrates significant improvements compared with state-of-the-art methods (GTB and EPSDC) on various metrics, such as the AUC, average precision (AP), and accuracy, particularly in terms of robustness and effectiveness in identifying potential drug combinations. As a result, it provides a more accurate and comprehensive framework for predicting drug-drug interactions (DDIs), demonstrating the potential of graph-based learning methods in the realm of pharmacovigilance and drug discovery.

Still, SeHNE focuses solely on drug pairs rather than higher-order combinations, due to the exponential increase in candidate space. Future research needs to develop strategies for narrowing this space to feasibly explore high-order drug combinations. Another limitation is that SeHNE primarily uses topological structures of heterogeneous networks, neglecting intrinsic drug attributes such as chemical structure and function. Incorporating these intrinsic features could improve the modeling and characterization of drugs. Additionally, SeHNE currently integrates only drug and protein information without considering gene expression, drug responses, or the immune microenvironment. Future work should aim to include these aspects to enhance the identification of effective drug combinations.

##### 3.4.1.2 CLDDI: a novel method for predicting drug-drug interaction events based on graph contrastive learning

Xu et al. (2023) use graph contrastive learning in their paper (Xu et al., 2023). Their proposed framework, named CLDDI, consists of three primary components: the contrastive learning module, the drug network structure embedding module, and the DDI prediction module. The contrastive learning module generates two graph views by randomly corrupting the original knowledge graph (KG) and maximizes the agreement of node representations between these views using a contrastive loss. These node embeddings are then used as initial representations in the drug network structure embedding module, which applies a graph convolutional neural network (GCN) to extract relational information from multi-relational DDI networks. The final drug embeddings are obtained by aggregating the embeddings from both modules and are used in the DDIp module to compute interaction-specific scores, combining contrastive and supervised loss for end-to-end learning. Experiments on real datasets demonstrate that CLDDI outperforms baseline methods in terms of AUC of 0.9923 and AUPR of 0.9886, showing significant improvements in accuracy, robustness, and generalization, particularly in sparse data scenarios.

Still, the study has some limitations, like the simplicity of the graph data augmentation techniques that were used, more specifically, the edge removal and node feature masking. More efficient and general methods for augmenting large, heterogeneous graph-structured data remain an open research direction. The issue of sparsity, not only in drug nodes but also in DDI events, with some events appearing infrequently in the dataset, can be noticed. Future research could address these unbalanced conditions by developing methods that can further enhance model performance in such scenarios. Key areas of investigation include the exploration of more advanced graph data augmentation techniques and addressing event sparsity.

##### 3.4.1.3 A model-agnostic framework to enhance knowledge graph-based drug combination prediction with drug-drug interaction data and supervised contrastive learning

Gu et al. (2023) applied supervised contrastive learning (SCL) in combination with biomedical knowledge graphs (KG) on DDI data to obtain drug combination predictions with high accuracy. Initially, the embeddings are extracted using various network embedding algorithms (random walk-based and graph neural networks). Then, pretraining is done with SCL to refine drug embeddings. Finally, a fully connected classifier is used for prediction. The DDIs from the TwoSides database were used as negative samples, which improved prediction accuracy through realistic negative examples, unlike randomly sampled drug pairs. The experimental results show significant performance improvements across multiple metrics for various algorithms, with the DREAMwalk algorithm showing the highest overall performance. DREAMwalk is a network embedding algorithm that can be used to generate embeddings from a knowledge graph. The visualization of embedding vectors using t-SNE and the robust performance in class-imbalanced settings are also evidence of the effectiveness of this framework.

Still, notable limitations are challenges in inferring combinations of drugs not included in the original KG. This issue can be mitigated by retraining the model with updated KGs that include new drugs and their relationships. Incorporating clinical knowledge, such as dosing plans and side effect severities, to enhance the clinical applicability of predictions could be a future research basis. Additionally, the framework could benefit from explicitly integrating disease entities into the prediction process, as current methods only implicitly use disease information. The study also suggests combining the strengths of KG-based and high-throughput screening (HTS)-based approaches to leverage both static, curated information from KGs and dynamic, cell-line-specific data from HTS.

##### 3.4.1.4 MAVGAE: a multimodal framework for predicting asymmetric drug-drug interactions based on variational graph autoencoder

Deng et al. (2024) introduced in 2024, the Multimodal Asymmetric Variational Graph Autoencoder (MAVGAE) framework that was designed to predict asymmetric DDI using a VGAE architecture where the encoder consists of a two-layer GCN. It integrates multimodal data, including drug molecular fingerprints, target interactions, enzyme interactions, and pathway information, and constructs a feature set for each drug. The network generates mean and standard deviation for the latent variables, capturing the relationships within the drug interaction network. The graph is reconstructed by the decoder with a vector dot product. The model was trained on a large-scale dataset with stratified subsets for training, validation, and testing. It was evaluated with metrics such as AUC, AUPR, accuracy, and F1-score. MAVGAE outperformed several benchmark methods, with a significant improvement in AUC and AUPR values by 2%–3% points. Including pathway, enzyme, and target data, along with the variational autoencoder component, significantly improves the model’s predictive performance, achieving an AUROC of 0.971, AUPRC of 0.964, and ACC of 0.913.

The model’s interpretability and dataset constraints are two main limitations in the current MAVGAE framework. The complexity and opacity of the variational graph autoencoder make it difficult to understand the model’s decision-making processes. This creates challenges for practical application and trust in the results. Furthermore, the restricted availability of datasets on asymmetric drug interactions limits the evaluation of the model’s performance, which has an impact on the overall assessment of its accuracy and generalizability. Enhancing the interpretability of the model to make its operations more transparent and developing richer, more diverse datasets to better evaluate and improve the model’s robustness and applicability in various contexts could represent future directions of research.

##### 3.4.1.5 MPHGCL-DDI: meta-path-based heterogeneous graph contrastive learning for drug-drug interaction prediction

Hu et al. (2024) predicts DDIs using a meta-path-based heterogeneous graph contrastive learning model, MPHGCL-DDI. The model contains two main contrastive views: an average graph view and an augmented graph view, constructed from multi-source drug information, including direct biological attributes and PPIs. The study uses three levels of data augmentation schemes—feature, edge, and sub-graph augmentation—to enhance the robustness and performance of the model. Unsupervised and supervised contrastive losses are combined along with a multi-relation prediction loss to optimize the training process. The MPHGCL-DDI model was evaluated on three prediction tasks across two datasets. It demonstrated superior performance over several models: MDDI-SCL (Multi-type Drug-Drug Interaction - Supervised Contrastive Learning) (Lin et al., 2022), MM-GANN-DDI (Multimodal graph-agnostic neural networks for predicting drug-drug interaction events) (Feng et al., 2023), MCFF-MTDDI (Multi-Channel Feature Fusion model for multi-typed DDI prediction) (Han et al., 2023), MP-DDI (Meta Path DDI) (Zhao et al., 2023), RaGSECo (Relation-aware graph structure embedding) (Jiang et al., 2024), by achieving higher accuracy, macro-F1, and other evaluation metrics. The macro-F1 is determined by calculating the F1 score independently for each class and then taking the average (without considering the class imbalance). It is particularly useful when all classes are equally important, regardless of their frequency. The MPHGCL-DDI approach addresses the challenge of class imbalance in DDI datasets and reveals latent drug relations through integrated biological information.

Some limitations of the MPHGCL-DDI model can be noticed. For example, the model tends to assign higher scores to DDI events with more instances due to the highly imbalanced distribution of instances across different DDI events. This issue suggests a need for further research on sampling methods and algorithmic models to address data imbalance, such as employing over-sampling methods for the minority class during model training. Additionally, the model demonstrates poor performance in predicting DDIs between two new drugs, which is useful in drug discovery. Future studies should prioritize resolving these ‘cold start’ challenges by focusing on the development of methods that can better handle new drug interactions.

#### 3.4.2 Matrix factorization methods

##### 3.4.2.1 Attribute supervised probabilistic dependent matrix tri-factorization model for the prediction of adverse drug-drug interaction

Zhu et al. (2021) used matrix factorization methods to decompose interaction matrices into lower-dimensional representations to predict unknown drug interactions. More specifically, it uses the Attribute Supervised Probabilistic Dependent Matrix Tri-Factorization (PDMTF) model. The PDMTF model incorporates two key drug attributes: molecular structure and side effects, alongside their correlation for effective adverse drug-drug interaction (ADDI) modeling. A probabilistic matrix tri-factorization approach was used. The adverse interaction matrix is decomposed into three matrices, capturing the predicted molecular structure and side effect matrices, and a dependent matrix to model drug dependencies. The optimization of PDMTF involves an alternating algorithm combining stochastic gradient descent (SGD) and alternating direction method of multipliers (ADMM). PDMTF outperforms several baseline models: SVMs, Multi-Dya (a multi-task dyadic regression model) (Jin et al., 2017), DLM (a deep learning method) (Ryu et al., 2018), BSNMF (a balanced semi-nonnegative matrix factorization model) (Shi et al., 2019), SFLLN (a sparse feature learning ensemble method with linear neighborhood regularization) (Zhang et al., 2019), PMF (classical probabilistic matrix factorization method) (Salakhutdinov and Mnih, 2007), CPMF (a context-aware probabilistic matrix factorization model) (Ren et al., 2017) and SC-PMF (Probabilistic Matrix Factorization with Social relationship and Content of items) (Tan et al., 2016) on evaluation metrics such as MAE, RMSE, and accuracy by achieving the lowest MAE and RMSE, and the highest accuracy. Specifically, PDMTF shows improvements of 23.67% in MAE compared to the classical probabilistic matrix factorization method and 15.06% and 9.79% improvements over its variants PDMTF-A and PDMTF-D in MAE, respectively.

Nevertheless, the paper notes the need for theoretical proof of the convergence of the optimization algorithm used in the PDMTF model. Second, it can be noticed that adverse drug-drug interactions are influenced by various attributes beyond molecular structure and side effects; hence, future work will involve incorporating more attributes and their correlation matrices into the model. Third, can be further investigated the pharmacogenetic and metabolic relationships among drugs to better understand the biological mechanisms underlying ADDIs. While the current work focuses on predicting whether a drug pair would lead to adverse interaction, it does not identify the specific types of adverse interactions. Future research should aim to predict detailed ADDIs, such as specific side effects like insomnia or gastrointestinal discomfort.

Table 5 displays a comparative overview of studies that employed structured learning techniques. Here are detailed the datasets, modeling strategies, and methods used together with the comparative analysis. The table highlights both matrix factorization and graph-based methods.

**TABLE 5:** Overview of the analyzed papers that use structured learning methods.

Method	Ref.	Article title	Method used	Comparative analysis	Comment
Graph based	Song et al. (2022)	Predicting combinations of drugs by exploiting graph embedding of heterogeneous networks	SeHNE (Semi-supervised Heterogeneous Network Embedding)	Superior AUC and average precision scores compared to state-of-the-art methods (e.g., gradient tree boosting, EPSDC)	The joint learning of feature extraction and prediction and insensitivity to classifiers leads to a stable performance across drug similarity measures
	Xu et al. (2023)	CLDDI: A Novel Method for DDIp Events Based on Graph Contrastive Learning	CLDDI (graph contrastive learning model)	Superior performance over baselines (e.g., Decagon, GMPNN-CS) in accuracy, F1 score, AUC and AUPR.	Strong generalization and robustness, especially on sparse datasets
	Gu et al. (2023)	A model-agnostic framework to enhance KG-based drug combination prediction with DDI data and SCL	Model-agnostic framework	Outperforms models that rely on random negative sampling, with better accuracy, precision, recall, and F1 score	Uses DDI data as a negative dataset and supervised contrastive learning for drug embedding vector optimization
	Deng et al. (2024)	MAVGAE: a multimodal framework for predicting asymmetric DDIp based on VGAE	MAVGAE (multimodal framework with Variational Graph Autoencoder)	Superior results in AUC, AUPR, and accuracy for asymmetric DDI prediction compared with DGAT-DDI and DGGAN.	Uses multimodal data sources and variational autoencoders for predicting non-symmetric drug interactions
	Hu et al. (2024)	MPHGCL-DDI: Meta-Path-Based Heterogeneous Graph Contrastive Learning for DDIp	MPHGCL-DDI (Meta-Path-based Heterogeneous Graph Contrastive Learning model)	Better performance than baseline models (MDDI-SCL, MM-GANN-DDI, MCFF-MTDDI, MP-DDI, RaGSECo)	Uses three levels of data augmentation strategies, which makes it effective at predicting rare DDI events
Matrix factorization	Zhu et al. (2021)	Attribute Supervised Probabilistic Dependent Matrix Tri-Factorization Model for the Prediction of Adverse Drug-Drug Interaction	Attribute Supervised PDMTF (Probabilistic Dependent Matrix Tri-Factorization) model	Outperforms eight baseline methods (e.g., matrix factorization-based models and ADDI prediction models)	Incorporates drug dependencies in ADDI prediction, and leverages molecular structures and side effect correlations

## 4 Discussion

Recently, ML techniques have been shown to be very promising for predicting DDIs. By looking at research performed in the last 6 years on predicting DDIs, we aimed to analyze the innovative methods and frameworks that were recently developed for predicting DDIs and find new avenues of research based on the limitations of the research studied.

The analyzed papers significantly contributed to DDI prediction. Integrating various data sources into predictive models is one of them. Studies have shown that combining chemical, biological, and phenotypic data can be very effective in creating rich feature sets used in ML models. This has led to more accurate and comprehensive DDIp. For instance, the use of multi-source drug information, including substructures, target interactions, side effects, and therapeutic relationships, has led to the construction of feature networks that significantly enhance prediction reliability.

The development and application of semi-supervised learning frameworks is another noteworthy contribution. These methods, particularly those employing (PU) learning and meta-learning techniques, have shown improvements in handling data sparsity and imbalance. These frameworks can generalize better and achieve higher predictive performance as they leverage both labeled and unlabeled data. One example is the implementation of the node2vec algorithm for network embedding, followed by bagging SVM classifiers. This can significantly improve prediction accuracy. Graph-based learning methods represent another significant advancement. GCNs and VGAEs have been shown to be very effective in modeling drug interactions. They can capture the complex relationships within heterogeneous networks, improving metrics such as AUC and AUPR. The introduction of graph contrastive learning enhances these models by maximizing the agreement between different graph views. This improves the model’s robustness and generalization, especially when the data is sparse.

Self-supervised learning and contrastive learning have introduced new ways to pre-train models on large datasets, creating robust molecular feature spaces. These methods have shown themselves to be competitive, particularly in predicting known drug interactions. They use data augmentation and batch balancing strategies to handle class imbalance.

Matrix factorization techniques, such as the PDMTF model, also offer efficient solutions for decomposing interaction matrices. These methods improve the prediction of ADEs by incorporating key drug attributes, such as molecular structure and side effects, alongside their correlations.

**FIGURE 5:**
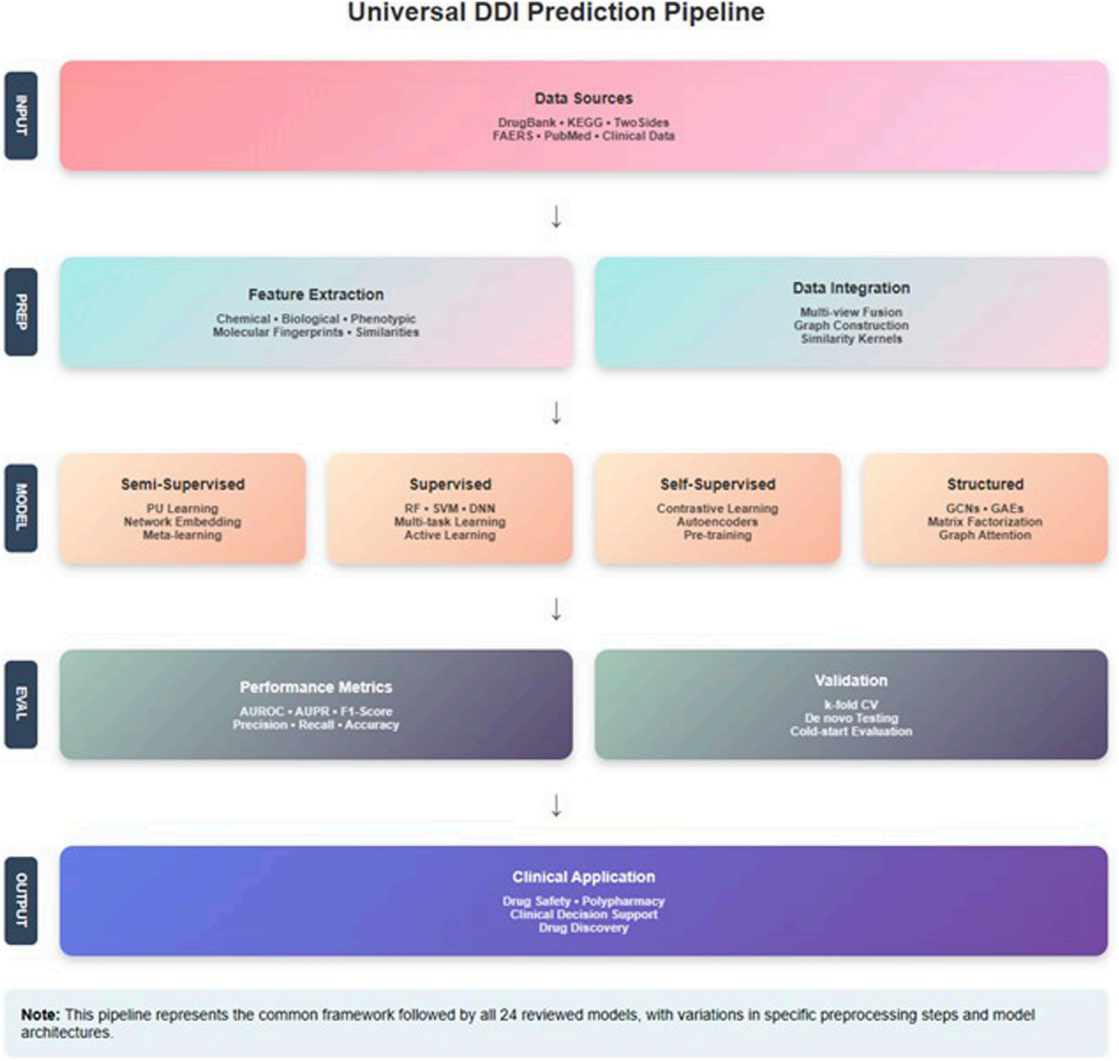
Universal pipeline for DDI prediction showing the typical workflow from input data to clinical application. This framework reflects common steps used across all 24 reviewed models, with some variation in preprocessing and modeling components.

The general structure followed by most DDI prediction methods is shown in Figure 5. It outlines the common steps from data input and feature extraction to model training and clinical use.

### 4.1 Comparative analysis of learning models for DDI prediction

Table 6 includes 24 models grouped by their learning type: supervised, semi-supervised, self-supervised, and structured, and it was created as a summary table to help compare the most important models discussed in this review. For each model, we show what data it used, what features it learned from, how well it performed (if performance was reported), and a few notes about its strengths or known issues. This overview gives a quick way to see how different approaches compare, and may help researchers choose the right method depending on their needs and data.

**TABLE 6:** Overview of 24 important DDI prediction models grouped by learning type. Includes data, features, performance, and main observations.

Learning type	Model (Ref.)	Data used (main)	Features used (core)	Reported performance^†^	Notes/limitations
Semi-Supervised	Meta-learning + PU SVM (Deepika and Geetha, 2018)	DrugBank + aux. networks	Node2Vec embeddings	+22% AUPR vs. LR/DT; +12.7% vs. 1-class SVM	Only relative gains reported; absolute metrics not given
	MLRDA (Chu et al., 2019)	C1IT, C2IS	Chem, indications, targets, SE	C1IT AUPR 0.440 (+0.10 over baselines); AUROC 0.667. C2IS AUPR 0.483; AUROC 0.697 ijcai.org	Multi-feature disentangling; small AUPR but largest relative lift
	Info-model TK-sim (Marushchak and Kosarevych, 2020)	DrugBank	Target + therapeutic sim	AUC 0.985 ± 0.05	Clustering step only 54% accurate
	Stacked-AE wSVM (Liu et al., 2020)	FAERS + DrugBank + Drugs.com	AE-derived AE-features	AUC: ≈ 0.868 F-measure: ≈ 0.816 Recall: ≈ 0.871 Precision ≈ 0.761	Relies exclusively on FAERS post-marketing reports. Peak gain occurs when ≤ 30% of data are labeled
	SeHNE (Tan and Ma, 2020)	Drug-drug sim. +DTI + PPI	NMF embeddings	AUC ≈ 0.70 (vs. 0.63 GTB)	Scalability constraints
	DDI-IS-SL (Yan et al., 2022)	DrugBank	Chem/bio/phenotype + GIP	5-fold AUC: 0.9691 10-fold: 0.9745; *de novo*: 0.9292	High but limited to similarity kernels
	Net-projection (Lv et al., 2024)	E.coli MG1655 DDI net (19 × 19) and BW25113 DDI net (68 × 68)	Spectral + hierarchical clustering → affinity matrix → semi-supervised graph projection	MG1655 — Acc 0.95, Prec 1.00, Rec 0.75, F1 0.86 BW25113 — Acc 0.81, Prec 0.88, Rec 0.64, F1 0.74	Pure topology (no chem/MoA); clusters correlate with MoA (ρ = 0.37); performance sensitive to σ ≥ 0.3 cut-off; scalability to large heterogeneous DDIs not yet shown
Supervised	TSSL SVM/GBM (Kastrin et al., 2018)	DrugBank KEGG NDF-RT TwoSides	Topological and semantic sims	AUPR 0.93 (RF/GBM)	Topology > semantics; weights ignored
	MTMA (Zhu et al., 2020)	DrugBank	Mol-struct + side-effect tensors	AUC 0.9247 AUPR 0.7515	AUPR still beaten by SFLLN.
	Active-Learning IR (Xie et al., 2021)	PubMed abstracts	TF-IDF + uncertainty	Precision ↑ 0.45 → 0.83 (SVM)/0.70 → 0.82 (LR)	Focus on retrieval, not DDI classif
	MDDI-SCL (Lin et al., 2022)	DrugBank	Attention + contrastive	AUPR 0.9782 Acc 0.9378 (task 1)	Weak on rare types/new drugs
	DeConDFFuse (Gupta et al., 2023)	BIOSNAP	Bioactivity signaturizer	AUC-ROC 0.897 AUPRC ↑ over KGNN	Higher FPs than Graph-DDI.
	PK-DDI SVR (Gill et al., 2023)	120 clinical studies	CYP450, f_m_, phys-chem	78% predictions within 2-fold exposure	Undershoots strong inhibitors
	CAGPool (Lee et al., 2023)	Decagon	Co-attention GNN	AUROC 0.872 AUPRC 0.832 AP@50 0.803	Pair-level graph pooling
Self-Supervised	MGP-DR (Ren et al., 2022)	DrugBank + unlabeled graphs	Mask-atom and SAB pre-tasks	RMSE 0.4009 MAE 0.4066	Only relative gains published
	DMVDGI (Zhuang et al., 2023a)	Decagon CRDs NCRDs	Multi-view graph	AUROC 0.9318 (Decagon) – beats HAN/GAT	View-choice and threshold sensitive
	ADGCL (Zhuang et al., 2023b)	Decagon + DrugBank	Heterog. signed network	↑AUROC and AUPRC over DeepDDI/KGNN	Time complexity high
	SMR-DDI (Kpanou et al., 2024)	ChEMBL22 (pre-train) + DrugBank	SSL CNN on SMILES	Balanced Acc 0.90 (task 1); ↓ on new-drug task (figures in pdf)	262-dim latent space limits expressiveness
	Dual-path GNN (Chandra Umakantham et al., 2024)	TwoSIDES DrugBank	VGAE pre-train	TwoSIDES: Precision 0.75, Acc 0.90 DrugBank Precision/F1/Acc: 0.99	Interpretability and multi-path stability
Structured	SeHNE (Song et al., 2022)	Hetero net (sim + DTI + PPI)	NMF + SVM	AUC ≈ 0.78 AP ≈ 0.55	Computationally time-consuming on large networks. Scalability and integration of additional drug attributes highlighted as future work
	CLDDI (Xu et al., 2023)	Multi-rel KG	Contrastive GCN	AUROC 0.9923 AUPR 0.9886	Strong on sparse events
	SCL + KG (Gu et al., 2023)	Multi-scale Interactome KG + DCDB 2.0 ≙ CDCDB + TWOSIDES	DREAMwalk embeds	Acc 0.923, Prec 0.946, Rec 0.898, F1 0.921	Needs re-training for unseen drugs. Uses real DDI negatives → robust to class imbalance
	MAVGAE (Deng et al., 2024)	Multimodal DDI graph	Variational GAE	AUROC 0.971 AUPRC 0.964 Acc 0.913	Black-box; asymmetric data scarce
	MPHGCL-DDI (Hu et al., 2024)	DrugBank + PPI	Meta-path contrastive	Known-known: Acc 0.9487/AUPR 0.9897 (D1); Acc 0.9541/AUPR 0.9927 (D2) Known-new: Acc 0.6872/AUPR 0.7208 (D1); Acc 0.6685/AUPR 0.6995 (D2) New-new: Acc 0.4634/AUPR 0.4227 (D1); Acc 0.4847/AUPR 0.4436 (D2)	Cold-start for new drugs. Handles rare events better via contrastive view
Matrix Fact	PDMTF (Zhu et al., 2021)	DrugBank	Mol + SE corr	MAE ↓ 23.7% vs. PMF; RMSE ↓ 15%	Needs convergence proof; cannot tell interaction type

^†^ Where authors gave multiple dataset splits; we list the headline/best value.

↑ Denotes a statistically significant improvement but no absolute figure in open text.

Looking at the table, we notice a few common patterns. Supervised models do well on training data, but they can have trouble when they are tested on new or noisy data. Semi-supervised models try to use both labeled and unlabeled data, but they work best with certain types of input. Self-supervised models are good when there is not much labeled data, but they are harder to understand. Graph-based methods and other structured models are good for showing how drugs interact with each other in complicated ways, but they are often harder to reproduce because they need a lot of data and settings. There are pros and cons to each type of model. The best choice depends on the data you have and whether accuracy, explainability, or scalability is more important.

**FIGURE 6:**
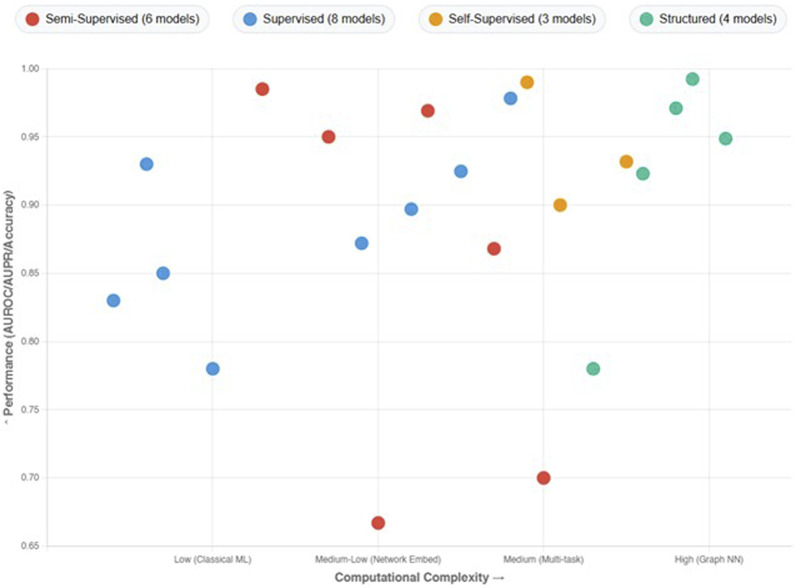
Performance versus computational complexity of the 24 reviewed DDI prediction models. Performance metrics from Table 6 plotted against estimated complexity scores, color-coded by learning paradigm.

It is helpful to look at how each model performs compared to how much computing power it needs. In Figure 6, are plotted the results from the papers we reviewed basically matching how well the models did with how demanding they are to run. The way we scored complexity was not exact, but more of a general estimate: simpler algorithms like RF or SVM were marked as low, and more advanced stuff like GNNs or multi-view learning ended up on the higher end.

Some patterns show up when you look at the plot. Sure, a lot of the time, the more complex models do better, but not always. A good example is DDI-IS-SL. It gets great results (around 0.97 AUROC) without needing a high amount of resources. That’s a good middle ground for people who want solid performance without using heavy computing power. There are models like MPHGCL-DDI or MAVGAE—top performers, but they also need serious horsepower. Meanwhile, standard supervised models tend to sit in the lower-left corner—not too heavy, pretty stable, but not outstanding either.

So, depending on what matters more—speed, accuracy, or just something that runs easily—this comparison gives a decent starting point for picking the right approach.

Key Insights from Real Performance Data:

• Top Performers: MPHGCL-DDI (0.95 AUROC) and MAVGAE (0.97 AUROC) lead in performance but require high computational complexity
• Efficiency Champions: TSSL achieves 0.93 AUPR with relatively low complexity (classical ML approach)
• Semi-Supervised Sweet Spot: DDI-IS-SL (0.97 AUC) and MLRDA show excellent performance-complexity balance
• Self-Supervised Promise: Limited data points, but SMR-DDI shows competitive performance with moderate complexity
• Complexity vs. Performance: Clear trend showing higher complexity often (but not always) correlates with better performance
• Method Selection: For resource-constrained environments, consider TSSL or classical supervised methods. For maximum performance, opt for structured methods like MPHGCL-DDI

**FIGURE 7:**
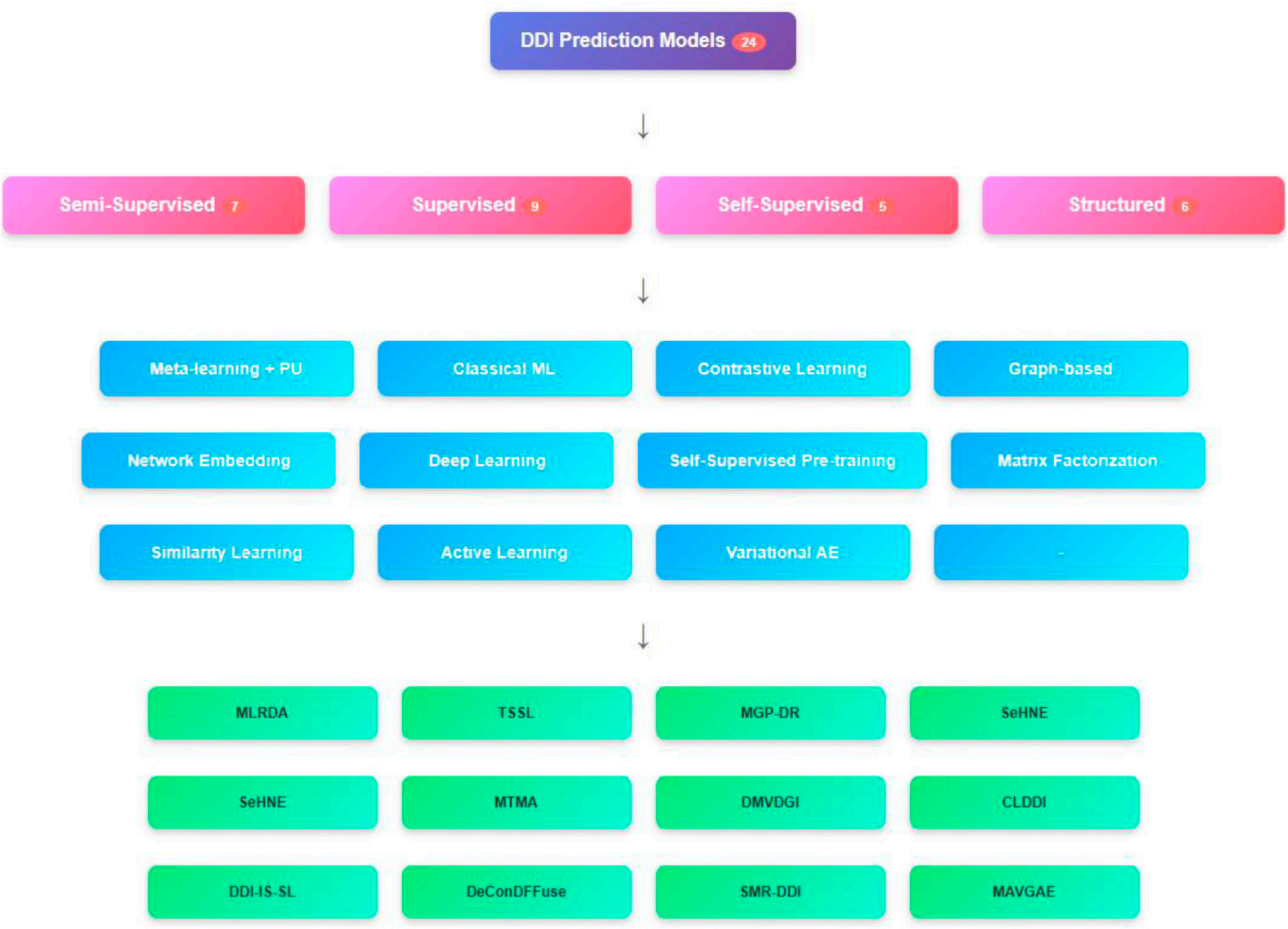
Taxonomy of the 24 reviewed DDI prediction models, grouped by learning paradigm (supervised, semi-supervised, self-supervised, structured) and showing their core methodological components.

To better illustrate the differences between the reviewed models, Figure 7 organizes them into categories based on their learning type and methodological approach.

### 4.2 Model recommendations and practical guidance

Different ML models have their strengths and trade-offs, and picking the right one really depends on what kind of data you have and what you’re trying to achieve. Some methods need lots of labeled data, while others can manage with less. A few are easier to interpret, which helps when you need to explain how the predictions were made. Others do better when the data is noisy or imbalanced. In Table 7, below, we’ve pulled together a simple overview to help compare these methods. It is not just about performance—it is also about what works best for your situation.

**TABLE 7:** Practical recommendations for choosing ML models in DDI prediction based on data constraints, interpretability, scalability, and reproducibility.

Method type	Best when data is limited	Good interpretability	Handles imbalance	Scales to large data	Reproducibility
Supervised	Needs labeled data	Yes (e.g., RF, linear)	Sensitive to bias	Yes (classical ML)	High (open tools)
Semi-Supervised	Works with few labels	Medium (graph embeddings)	Some use PU learning	Varies by graph model	Often underreported
Self-Supervised	Excellent for low labels	Limited (autoencoders)	Contrastive helps	Yes (scales well)	Often no code/docs
Structured (GNN/MF)	Needs full graph/data	Often black-box	GCNs resist imbalance	Slower on large graphs	Reproducibility varies

**FIGURE 8:**
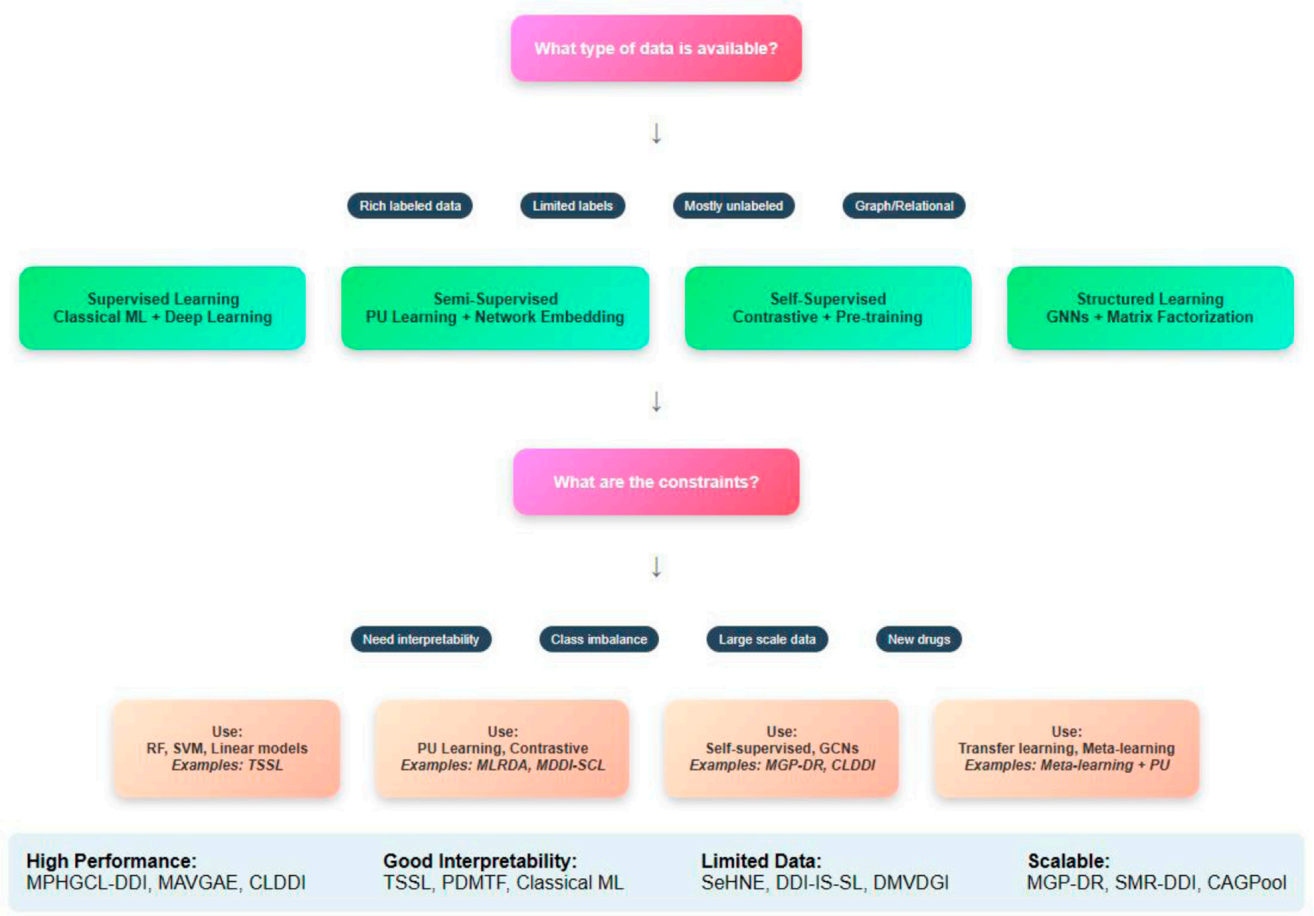
Decision guide for selecting appropriate DDI prediction approaches based on data characteristics and computational or interpretability constraints.

Figure 8 provides a practical guide to choosing a suitable model based on data availability and constraints such as scalability, interpretability, or the presence of class imbalance.

### 4.3 Common challenges and limitations

Data imbalance is the most common challenge across studies. Many datasets used in DDIp are disproportionate, because most of the data consists of negative interactions (no interaction) and only a few are positive interactions (actual DDIs). This imbalance causes models to become biassed toward the majority class, so lowering their sensitivity and precision in spotting real positive interactions. This is especially apparent in the underperformance of models trying to forecast rare or novel DDIs. In this regard, strategies including advanced methods like PU learning, under-sampling the majority class, or oversampling the minority class are usually advised to minimize this issue.

Many studies mostly rely on particular databases including DrugBank, KEGG, and FAERS, which might have only few DDIs. Models taught just from DrugBank data may overlook interactions reported in other databases or in the real-world clinical environment. This reduces the generalizability of the outcomes since the models might not perform as well on unprocessed data coming from many sources. EHRs and other clinical databases could help to boost model resilience and augment the data sources.

Reproducibility and methodological transparency remain consistent challenges across many of the reviewed studies. Although most models are developed using publicly available datasets, efforts to replicate results are frequently limited by the absence of shared source code, detailed training configurations, and clearly documented hyperparameter settings. Consequently, this review relies solely on the information explicitly reported within the articles themselves—namely, in the main text, figures, and appendices. While this restricts the ability to independently validate reported outcomes, it also highlights a broader issue within the field. Encouragingly, some recent works have begun to embrace more open research practices, offering public repositories and clearer methodological documentation. Moving forward, greater emphasis on open-source implementations, standardized reporting, and reproducible workflows would substantially improve the reliability and comparability of DDI prediction models.

DL and graph-based approaches are advanced models that often demand large computing capability and long training times, which limits their application in real-world clinical environments. Their complexity makes them difficult to understand as well; decisions in medicine are rarely clear-cut, which erodes confidence and acceptance. Still, the key is to improve interpretability and efficiency.

A common situation in drug development, many models also battle unseen or understudied drugs. Usually, this results from overreliance on known interactions and limited labeled data. In such situations, techniques like transfer learning could help to improve generalization.

The choice of negative samples raises still another problem. Random pairing may cause skew results and mislabeling of data. Both model accuracy and stability could be enhanced by active learning—that is, by using smarter sampling techniques.

These difficulties taken together affect the DDI prediction tool’s dependability and applicability. To get these approaches toward more general clinical use moving forward, better data, simpler models, and smarter training pipelines are needed.

### 4.4 Future research directions and proposed improvements

Based on the limitations of the actual researches, future researches should prioritize the integration of diverse and comprehensive data sources, improving the robustness and generalizability of DDI prediction models. One idea would be to incorporate the EHR, real-world evidence, and additional clinical databases alongside existing sources like DrugBank and KEGG. In this manner, researchers can create more holistic models that capture a wider range of DDIs. Deep insights into the biological mechanisms underlying DDIs can be obtained by also integrating genomic, proteomic, and metabolomic data.

Future studies should explore advanced sampling and data augmentation to address data imbalance. Techniques such as synthetic minority oversampling (SMOTE) and generative adversarial networks (GANs) can be used to generate synthetic data for underrepresented classes, thereby balancing the dataset. Refinement of training data can also be done using active learning strategies, which iteratively select the most informative samples for labeling, with the scope to improve the model performance on imbalanced datasets.

Managing the interpretability of complex models, including DL and graph-based approaches, can help to be adopted in clinical practice. Next studies should try to produce more interpretable models and apply methods that make the decision-making process of these models more transparent, also, should include explainable artificial intelligence (XAI) frameworks, feature importance analysis, and attention mechanisms to help to clarify how forecasts are produced.

Without sacrificing performance, methods including model pruning, quantization, and the use of more efficient neural network architectures—e.g., transformers—can greatly improve computational efficiency linked with advanced ML techniques. Real-time applications can find these models more accessible if improvements lower training times and computational resource needs.

The ability of models to generalize to new or less-studied drugs makes them useful in drug discovery and development. Future studies should explore transfer learning and continual learning approaches that allow models to adapt to new data incrementally. Techniques should be improved to learn from small amounts of data, such as few-shot learning and meta-learning, which can also help models predict interactions for unknown compounds more accurately.

Prediction model performance depends heavily on the selection of negative samples. Developing more informed and accurate methods for negative sample selection is another avenue that can be followed in future research. Using high-confidence negative samples allows the models to iteratively enhance the training set, thereby raising the general quality of the training data. Furthermore, assisting in the choice of more biologically plausible negative samples is domain knowledge.

Advancing DDIp would greatly benefit from other learning frameworks, including self-supervised learning, contrastive learning, and hybrid models combining several learning paradigms. Large unlabeled datasets can be used to pre-train models, which are then fine-tuned on labeled data, creating robust and high-performance models. These frameworks can be researched further.

Other clinical knowledge, such as dosing regimens, patient demographics, and comorbidities, could also be included in DDIp models, which might improve their practical applicability. Next studies should aim to integrate these factors to provide more contextually relevant predictions. Additionally, developing models that can account for the dynamic nature of drug interactions, such as time-dependent effects and varying drug response profiles, can further improve the clinical relevance of predictions.

## 5 Conclusion

### 5.1 Semi-supervised learning models

Using both labeled and unlabeled data, semi-supervised learning methods explored have shown great promise in DDIp. These methods enhanced the models’ performance in situations with few annotations. Data sparsity and imbalance have been sufficiently addressed by methods including: network embedding, meta-learning, and PU learning. As mentioned, reproducibility remains difficult since many studies lack open-source code, thorough hyperparameter documentation, and consistent dataset partitioning. To improve the replication of the model so it can be compared across studies, future studies should give methodological transparency, clear benchmarking procedures top priority. Open-access implementations are of secondary importance.

### 5.2 Supervised learning models

Strong predictive performance in DDIp has come from both DL architectures and supervised ML techniques, including conventional models like RF and SVMs. The availability of well-labeled datasets determines the general efficacy of these models; while beneficial, their applicability in real-world situations when such labels are rare is limited. Furthermore, adding to reproducibility difficulties are dataset variability, variations in feature engineering, and inconsistent preprocessing methods. Adoption of supervised models in clinical decision support systems needs standardized evaluation systems, better model interpretability, and computational efficiency enhancements.

### 5.3 Self-supervised learning models

By decreasing the demand for large-scale labeled datasets in order to enhance molecular representation learning, these models have developed into a promising paradigm for DDIp. Variational autoencoders and contrastive learning, along with other graph-based techniques, highlight the capability to capture complex drug relationships. However, many of these studies lack meticulous statistical validation. This makes it harder to evaluate the applicability of the stated results of each research. The lack of open-source implementations and clear experimental settings makes it more challenging to replicate each study. Furthermore, a scalability provocation is the computational inefficiency. To achieve a balance between predictive performance and computational costs, future studies should prioritize the adoption of: benchmark datasets, open methodological reporting, and algorithmic optimizations.

### 5.4 Structured learning models

Graph-based and matrix factorization techniques, as well as other structured learning models, have given fresh ideas for drug interaction prediction and representation. Though they call for large computational resources, GCNs and contrastive learning approaches have improved drug representation learning. Although matrix-based techniques increase model interpretability, they sometimes require large amounts of data preprocessing, which can cause variations across studies. Establishing standardized protocols for statistical validation, guaranteeing open-access documentation, and investigating optimization strategies that lower computational complexity yet preserve predictive accuracy will help to forward structured learning in DDI research.

### 5.5 General conclusion

DDI prediction has made major progress through advances in feature representation, data integration, and accuracy. Reliable interaction modeling has resulted from methods including network embedding, GNN, and ensemble learning. Common difficulties still exist, though, in all learning paradigms, including data imbalance, computational efficiency limits, and model interpretability. Meaningful cross-study comparisons depend on open-access datasets, transparent methodologies shared, and consistent evaluation metrics guaranteeing reproducibility.

Improving model robustness using data integration from several biological, chemical, and clinical sources should be the main emphasis of the next studies. Furthermore, techniques like continuous learning and transfer learning can enable models to better generalize to hitherto unmet drug interactions. Using their integration into clinical decision support systems (CDSSs), these predictive models will be much appreciated in their practical application since they will help healthcare professionals to make more informed prescribing decisions, reduce adverse drug interactions, and maximize therapeutic strategies. Addressing these challenges will help bridge the gap between computational models and clinical use.
